# Optimal fishing effort benefits fisheries and conservation

**DOI:** 10.1038/s41598-021-82847-4

**Published:** 2021-02-15

**Authors:** Adam Rees, Emma V. Sheehan, Martin J. Attrill

**Affiliations:** School of Biological and Marine Sciences (Faculty of Science and Engineering), University of Plymouth, Drake Circus, Plymouth, PL4 8AA UK

**Keywords:** Environmental impact, Conservation biology, Ecosystem ecology, Ecology, Biodiversity

## Abstract

The ecosystem effects of all commercial fishing methods need to be fully understood in order to manage our marine environments more effectively. The impacts associated with the most damaging mobile fishing methods are well documented leading to such methods being removed from some partially protected areas. In contrast, the impacts on the ecosystem from static fishing methods, such as pot fishing, are less well understood. Despite commercial pot fishing increasing within the UK, there are very few long term studies (> 1 year) that consider the effects of commercial pot fishing on temperate marine ecosystems. Here we present the results from a controlled field experiment where areas of temperate reef were exposed to a pot fishing density gradient over 4 years within a Marine Protected Area (MPA), simulating scenarios both above and below current levels of pot fishing effort. After 4 years we demonstrate for the first time negative effects associated with high levels of pot fishing effort both on reef building epibiota and commercially targeted species, contrary to existing evidence. Based on this new evidence we quantify a threshold for sustainable pot fishing demonstrating a significant step towards developing well-managed pot fisheries within partially protected temperate MPAs.

## Introduction

Commercial bottom-towed fishing methods (such as trawling and dredging) are regarded as the most damaging to seabed habitats, with extensive direct and indirect effects on sensitive epifauna, such as temperate reefs^1–3^. This has led to bottom-towed fishing often being excluded from within some Marine Protected Areas (MPAs), including off England’s coast, to protect discrete patches of seabed from damage or disturbance^4,5^. MPAs that restrict activities known to be damaging often permit other potentially less-impactful activities to continue within them, e.g. alternative commercial fishing methods; these are commonly referred to as ‘partially protected’ MPAs^6–10^. Well-managed commercial fisheries are central to ensuring a partially protected MPA provides effective conservation of its resources and management of permitted activities^11–19^. For different fishing methods allowed within partially protected MPAs, the quantification of fishing intensity thresholds relating to all ecosystem components, including seabed integrity, target and non-target fishery species, would be useful when implementing adaptive management for commercial fishing activities^18,20,21^.

Restrictions on mobile fishing methods (e.g. bottom-towed fishing methods) inside MPAs can also reduce conflict between different marine users and provide new opportunities for alternative fishing methods, such as static gear fisheries (e.g. commercial pot fishing for crabs and lobsters)^22^. In England, many high-value species targeted by such fisheries are generally not subject to quota limitations and management is typically less restrictive^23,24^. The majority of English inshore MPAs permit static methods of commercial fishing to continue and expand on account of their impacts being considered benign^25,26^. Despite the extent and ubiquity of this fishing method, there are very few primary evidence sources that adequately address the ecosystem effects of pot fishing in temperate marine systems^25–29^. Direct physical effects associated with pot fishing (crushing, scouring, abrasion) are, however, conceivable, particularly during repeated deployment and hauling^30^. The conclusion from this limited literature is that pot fishing has no discernible effect on seabed epifauna. These studies, however, are limited in their duration and experimental design as they consider neither the effects at the ecosystem level nor the potential effects of over-exploiting their target fisheries, often leading to ambiguous conclusions^25,26,29^. It is therefore feasible that intensive commercial pot fishing may be having undocumented effects on temperate seabed habitats and target fishery species, which could compromise the conservation ambitions of partially protected MPAs implemented for their protection.

**Figure 1:**
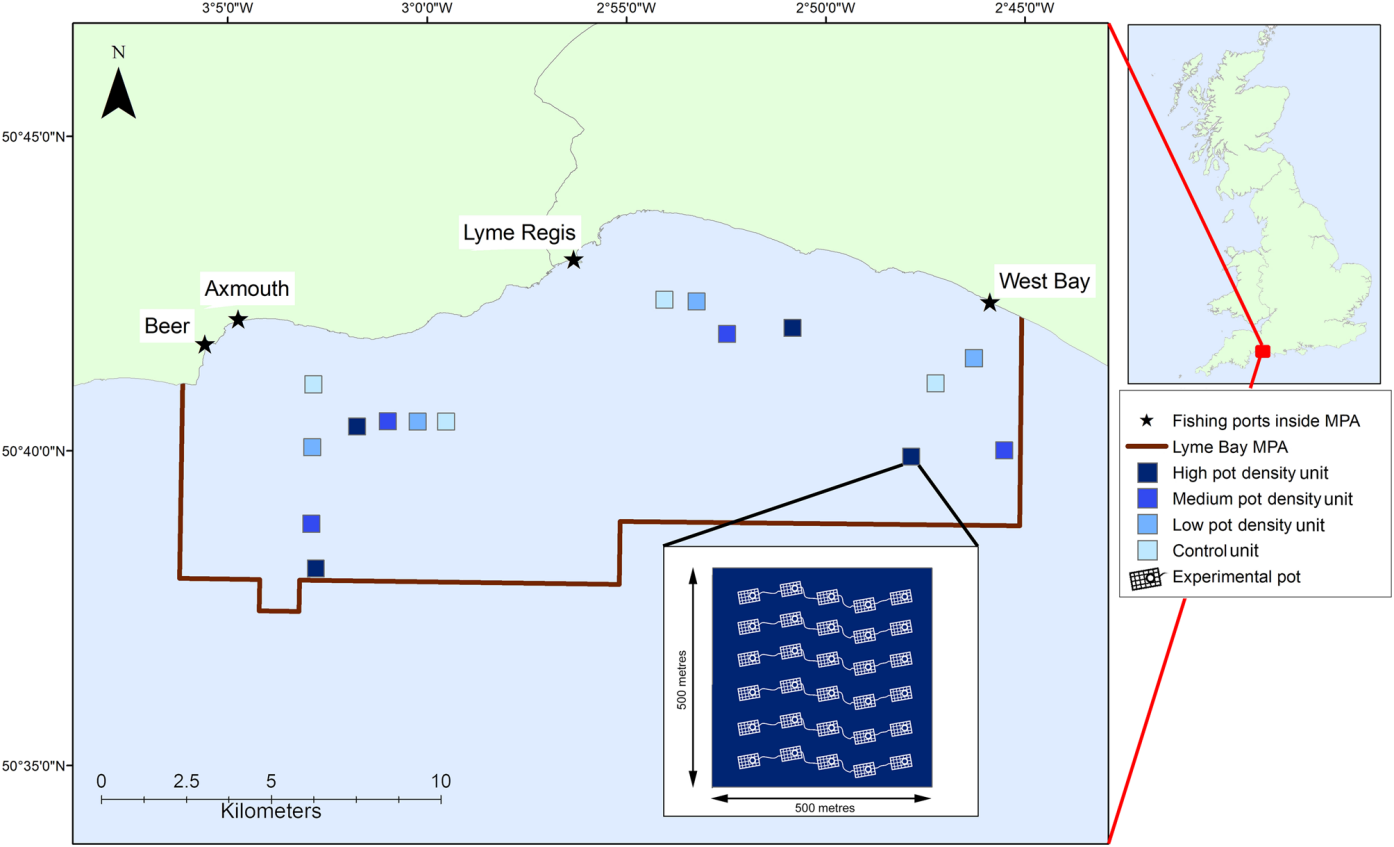
Lyme Bay Marine Protected Area (MPA) and experimental pot fishing study design. Locations of 16 experimental pot density treatment units [Control (No), Low, Medium, High] represented by different (blue) shaded squares inside the Lyme Bay MPA (brown line). Sets of four treatment units are aggregated into ‘areas’. Fishing ports involved in the study are labelled. Schematic example of a High pot density unit is shown; this map was created using the software ArcGIS version 10.7; https://desktop.arcgis.com/en/arcmap/.

The aim of this present study was to assess the ecosystem effects of pot fishing effort (density of pots targeting crab and lobster) on temperate reef building and reef associated organisms of both conservation and commercial importance. We performed an extensive and unprecedented experimental field study, carried out in partnership with local fishermen, within the partially protected Lyme Bay MPA (SW England), previously closed to bottom-towed fishing. We exposed units (500 m × 500 m) of reef to different levels of pot fishing effort (no pots, low, medium and high densities of pots, Fig. 1) over a sustained period of 4 years. This gradient in fishing intensity allowed us to identify the threshold density level above which effects occurred over an appropriate time period to assess change of target species’ metrics and the wider benthic community^31,32^. A combination of underwater video and extractive survey methods were employed to monitor the effects of an increasing density of pots on recovering benthic reef epibiota. The video data were used to assess Taxon Richness, Abundance and Indicator species from three functional groups: ‘Reef Builders’, ‘Sessile and Sedentary Reef Associates’ and ‘Mobile Reef Associates’. Data from experimental pot fishing surveys were used to assess the abundance, size and weight of the fishery target species brown crab (*Cancer pagurus*) and European lobster (*Homarus gammarus*).

Contrary to existing evidence, we demonstrate for the first time negative effects of pot fishing on both the benthic epibiota and the fishery, and that this effect is dependent on pot density. After 4 years, lower numbers of some key Reef Builders were observed in units subjected to a higher density of pots. This high fishing effort also led to a reduction in the quality and quantity of commercially targeted species. We also determine, importantly, that a pot fishing intensity ‘threshold’ exists and therefore commercial pot fisheries can be compatible with management plans inside MPAs when maintained at low, sustainable levels often observed by small-scale inshore fishers. The evidence presented here demonstrates a significant step towards informing and developing well-managed pot fisheries inside partially protected MPAs, underpinned by an ecosystem approach to management^12^.

## Results

### Reef builders and reef associates

**Figure 2:**
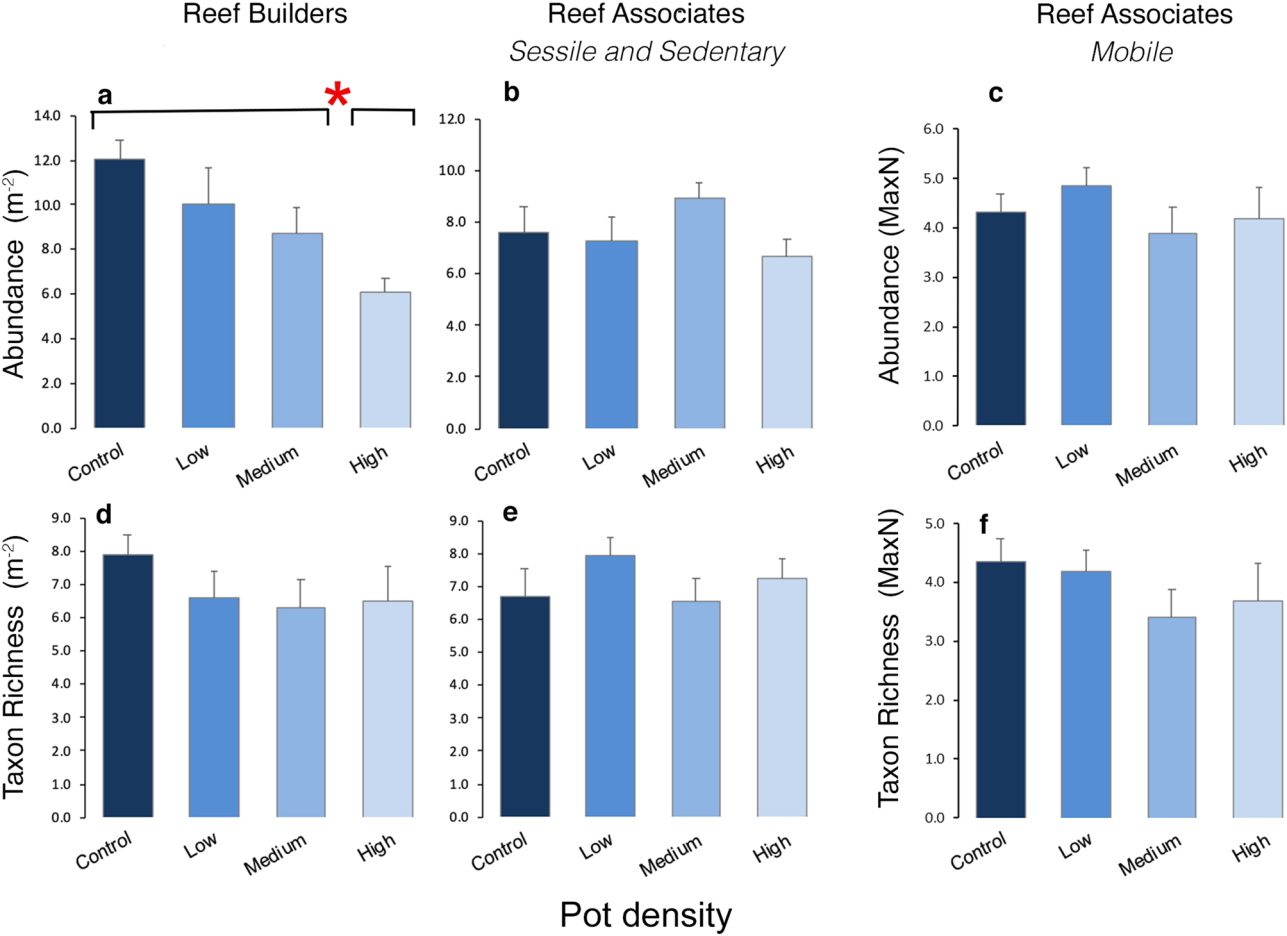
Response variables for each functional group by pot density treatment. (**a**–**c**) mean Abundance and (**d**–**f**) Taxon Richness (PERMANOVA main and pairwise results from Table 1, Supplementary Table 2), + standard error of the mean, for (**a**,**d**) grouped Reef Builders, (**b**,**e**) Sessile and Sedentary Reef Associates and (**c**,**f**) Mobile Reef Associates, for each pot density treatment. Main test significance (*P* ≤ 0.05) is denoted with red asterisk and black lines above bars identify where differences between Treatments occur.

Following 4 years of sustained pot fishing effort a significant Year × Treatment interaction (Table 1a, PERMANOVA, *F* = 1.228, *P* = 0.0147) for the response variable Abundance of Reef Builders group was observed. Pairwise comparisons indicated that in 2014 there was no difference between treatments, but in 2017 Control, Low and Medium treatments all differed to the High treatment (P > 0.05) (Fig. 2a, Table 1b). Mean Abundance of Reef Builders in the High treatment was 36% lower (4.16 indv. m^−2^ ± 0.131) compared to the other treatments (10.06 indv. m^−2^ ± 0.93). However, the Taxon Richness of Reef builders remained consistent and similar between all treatments (Fig. 2d, Supplementary Table S2). The Abundance and Taxon Richness of the Reef Associates (Sessile and Sedentary, Mobile) were consistent and similar across all pot density treatments (Fig. 2b,c,e,f, Supplementary Table S2; PERMANOVA tests all > P 0.05).

**Table 1:** **a** PERMANOVA main test on fourth-root transformed data for differences in Abundance of Reef Builders between factors Year (fixed: 2014, 2017) Treatment (fixed: Control (No), Low, Medium, High) and Area (random: Axmouth, Beer, Lyme Regis, West Bay) and **b** pairwise comparisons Year × Treatment significant interactions.

(**a**)	Group	Response variable	Model				
	Reef Builders	Abundance	Source	df	SS	Pseudo-*F*	*P* (perm)
			Year (Ye)	1	489.51	2.4928	**0.0086**
			Treatment (Tr)	3	1446	4.335	**0.028**
			Area (Ar)	3	294.43	0.6178	0.6372
			Ye × Tr	3	6440.37	1.228	**0.0147**
			Ye × Ar	3	589.12	1.4774	0.2784
			Tr × Ar	9	370.18	1.2764	0.2378
			Residual	105	42,045		
			Total	127	51,675		

(**b**)	Pairwise comparisons	2014		2017			
	Groups	t	*P* (perm)	t	*P* (perm)		
	No, Low	1.5605	0.2038	0.36158	0.7278		
	No, Med	0.78834	0.4809	0.99654	0.3481		
	No, High	0.78769	0.4924	2.8334	**0.0043**		
	Low, Med	0.74809	0.5237	0.55338	0.5929		
	Low, High	0.6875	0.7214	2.4804	**0.019**		
	Med, High	1.1256	0.314	3.4912	**0.0108**		

Significant results are displayed in bold (*P* (perm)). Degrees of freedom (df), Sum of Squares (SS) and *F* are reported.

**Figure 3:**
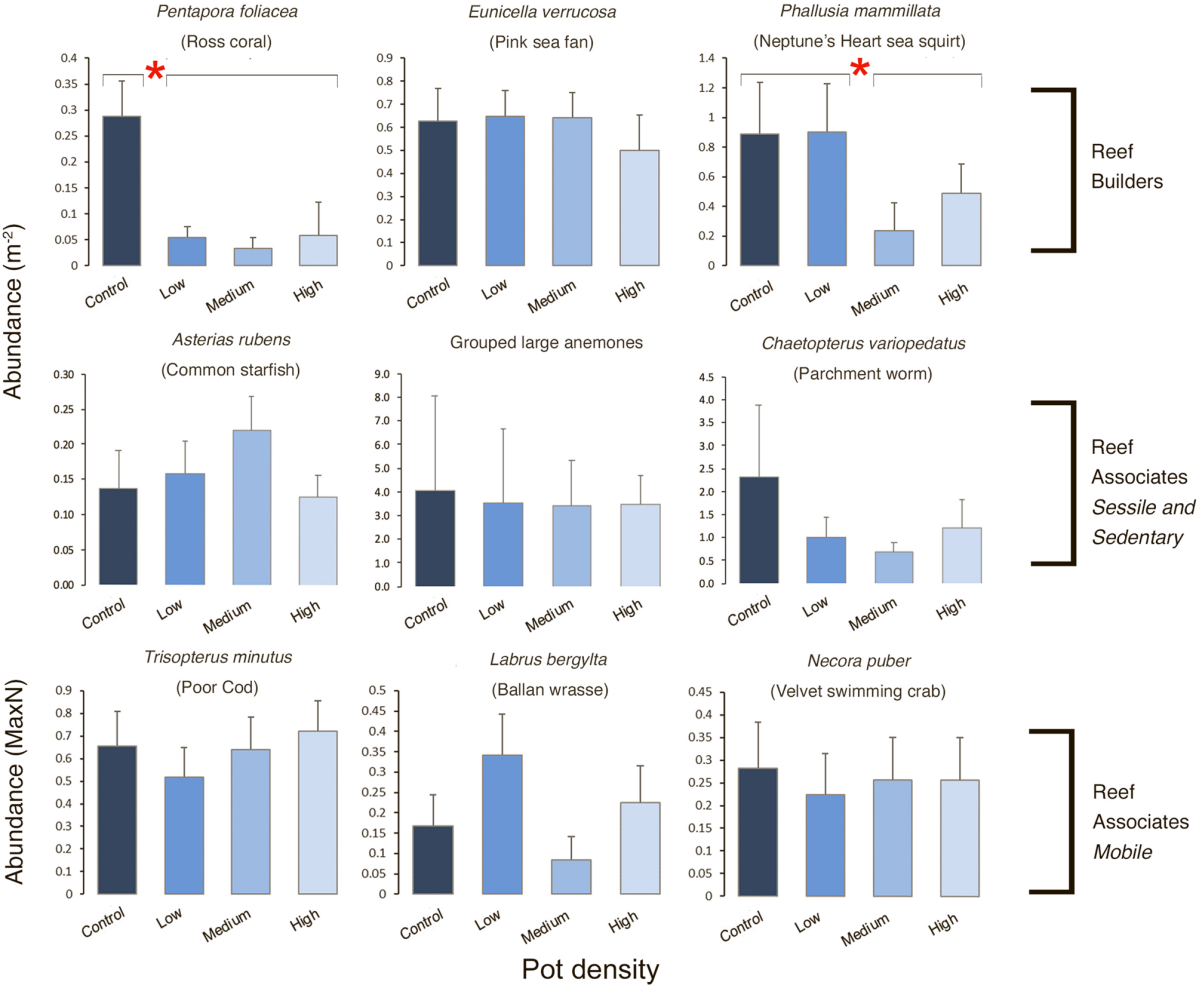
Abundance of Indicator taxa by pot density Treatment. Mean Abundance (PERMANOVA main and pairwise results from Table 2, Supplementary Table 3) + standard error of the mean, in each pot density Treatment, for nine Indicator taxa (three per Group). Main test significance (*P* ≤ 0.05) is denoted with red asterisk and black lines above bars identify where differences between Treatments occur.

Of the nine indicator taxa assessed, a significant Year × Treatment interaction was observed for two taxa, both of which were from the Reef Builders group (Fig. 3): the Ross coral (*Pentapora foliacea*) (Table 2, PERMANOVA, *F* = 2.383, *P* = 0.021) and the Neptune’s Heart sea squirt (*Phallusia mammillata*) (Table 2, PERMANOVA, *F* = 4.995, *P* = 0.045). Pairwise comparisons indicated that in 2014 there were no differences between treatments in the Abundance of either of these taxa. However, in 2017 abundances of *P. foliacea* were significantly different between the Control and all other treatments (P > 0.01) (Fig. 3, Table 2), while abundances of *P. mammillata* in the Control and Low treatments both differed to Medium and High treatments (P > 0.05) (Fig. 3, Table 2). Abundances of *P. foliacea* were significantly lower across all potted treatments (Low, Medium, High) in comparison to the Control (Fig. 3, Table 2, PERMANOVA: *F* = 5.9, *P* = 0.124). The mean Abundance of *P. foliacea* was 83% greater in the Control (0.287 indv. m^−2^) compared to the potted treatments (0.048 indv. m^−2^)*.* Abundance of *P. mammillata* was significantly lower in the Medium (0.23 indv. m^−2^) and High (0.49 indv. m^−2^) treatments in comparison to the Low treatment (0.9 indv. m^−2^) and Control (0.89 indv. m^−2^) (Fig. 3, Table 2, PERMANOVA: *F* = 3.86, *P* = 0.0133), a mean difference of 74% (Control and Low vs Medium and High). Although not found to be significant (Supplementary Table S3, PERMANOVA: *F* = 1.729, *P* = 0.239), Abundance of the Reef Building Indicator Pink sea fan (*Eunicella verrucosa*) was lowest in the High treatment indicating a similar response (Fig. 3). Of the Sessile and Sedentary Reef Associate Indicators no discernable trend was observed for the common starfish (*Asterias rubens*) or the grouped large anemones, both sedentary, however, the only sessile Reef Associate tested, the tube building Parchment worm (*Chaetopterus variopedatus*), did show a declining trend in Abundance with increasing pot density but this was not found to be significant (Fig. 3, Supplementary Table S3, PERMANOVA: *F* = 3.89, *P* = 0.098). None of the Mobile Reef Associate Indicators showed any significant Treatment differences and no clear trends were observed (Fig. 3).

**Table 2:** PERMANOVA main test (and pairwise comparisons) on fourth-root transformed data for differences between factors Year (fixed: 2014, 2017), Treatment (fixed: Control (No), Low, Medium, High) and Area (random: Axmouth, Beer, Lyme Regis, West Bay) for the response variable Abundance for two Indicator taxa; *Pentapora foliacea* and *Phallusia mammillata*.

**Indicator**	Response variable	Model					Pairwise comparisons	2014		2017	
	Abundance	Source	df	SS	Pseudo*-F*	*P* (perm)	Groups	t	*P* (perm)	t	*P* (perm)
***Pentapora foliacea***											
		Year (Ye)	1	2.0565	34.517	**0.029**	No, Low	1.5667	0.4337	3.4297	**0.0019**
		Treatment (Tr)	3	1.0293	5.983	0.057	No, Med	0.7746	0.7198	3.8006	**0.0004**
		Area (Ar)	3	0.7018	2.0836	0.171	No, High	1.5667	0.4344	3.1717	**0.003**
		Ye × Tr	3	1.0105	2.383	**0.021**	Low, Med	1.9283	0.3784	0.76131	0.5433
		Ye × Ar	3	0.1787	1.2645	0.331	Low, High	1.329	0.3278	0.2987	0.8015
		Tr × Ar	9	0.5894	1.3899	0.209	Med, High	0.3282	0.128	0.95988	0.3821
		Residual	105	5.04							
		Total	127	10.606							
***Phallusia mammillata***											
		Year (Ye)	1	10.829	293.4	**0.0016**	No, Low	1.7193	0.2795	0.1616	0.8837
		Treatment (Tr)	3	0.7497	3.575	**0.0197**	No, Med	0.8329	0.2378	2.5303	**0.0142**
		Area (Ar)	3	0.846	1.6452	0.2498	No, High	0.9213	0.4382	2.571	**0.0131**
		Ye × Tr	3	0.7884	4.955	**0.045**	Low, Med	0.236	0.7382	2.4665	**0.0175**
		Ye × Ar	3	0.1107	0.528	0.6604	Low, High	0.4578	0.7697	2.4534	**0.01601**
		Tr × Ar	9	0.5427	2.452	0.135	Med, High	0.3764	0.7702	1.4899	0.1636
		Residual	105	10.186							
		Total	127	24.055							

Significant main and pairwise test results are displayed in bold (*P* (perm)). Degrees of freedom (df), Sum of Squares (SS) and *F* are reported.

### Commercial fishery

**Figure 4:**
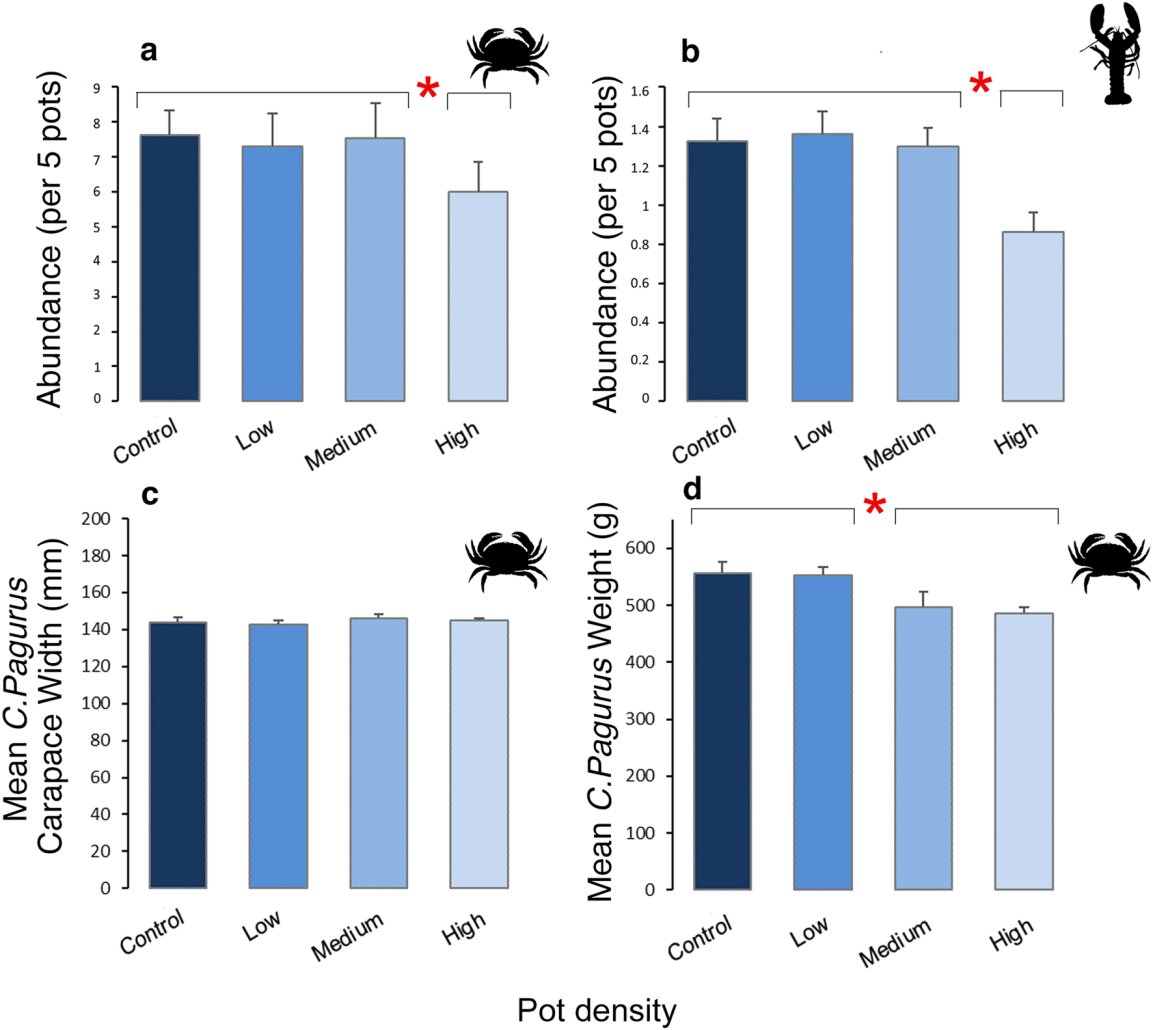
Abundances of commercially targeted species, and brown crab (*Cancer pagurus*) biometric response variables, by Treatment (pot density). (**a**,**b**) Mean Abundance (PERMANOVA main and pairwise results from Table 3), + standard error of the mean, for (**a**) *Cancer pagurus* (brown crab) [Control (No): n = 609, Low: n = 583, Medium: n = 602, High: n = 481] and (**b**) *Homarus gammarus* (European lobster) [Control (No): n = 106, Low: n = 109, Medium: n = 104, High: n = 69] by pot density treatment. (**c**) Mean *C. pagurus* Carapace Width and (**d**) mean *C. pagurus* Weight by pot density treatment (+ standard error of the mean). Main test significance (*P* ≤ 0.05) is denoted with red asterisk and black lines above bars identify where differences between Treatments occur.

Following 3 years of controlled pot fishing a significant Year × Treatment interaction indicated differences in Abundance between treatments varied over time for both commercially targeted brown crab (*Cancer pagurus*) (PERMANOVA: *F* = 3.4078, *P* = 0.028) and European lobster (*Homarus gammarus*) (PERMANOVA: *F* = 3.2738, *P* = 0.0484) (Fig. 4a,b, Table 3a). Pairwise comparisons showed no difference between treatments in Abundance for both *C. pagurus* and *H. gammarus* in 2014, however, in 2017 Abundance was significantly lower in the High treatment in comparison to all other treatments (P < 0.05, Table 3a) for both *C. pagurus* and *H. gammarus.* In the High treatment, it was found that, on average, Abundance was 19% (1.46 indv. per 5 pots) and 35% (0.46 indv. per 5 pots) lower for *C. pagurus* and *H. gammarus,* respectively*,* when compared to the other treatments (Fig. 4a,b). In addition, for *C. pagurus* individuals, a Year × Treatment interaction for the response variable Weight indicated treatment differences varied significantly over time (Table 3b, PERMANOVA: *F* = 6.694, *P* = 0.124). Pairwise comparisons showed no difference in Weight between treatments in 2014, while in 2017 mean Weight differed significantly between the Medium and High treatments and the Control and Low treatments (P < 0.05, Table 3b), a mean decrease of 35 g per individual (7%) in weight (Fig. 4d). This was not related to a change in individual size, as mean Carapace Width remained consistent and similar (Table 3b) across all treatments (Fig. 4c). The lower weights observed therefore suggests that the condition of *C. pagurus* individuals caught inside of Medium to High density treatments is a consequence of increased pot fishing, affecting the overall quality of the catch. For *H. gammarus,* mean Weight and Carapace Length did not show a response to different pot density treatments (Table 3b). Our results therefore suggest that the condition of *H. gammarus* was not affected by varying pot density.

**Table 3:** PERMANOVA main test results (and pairwise comparison results for significant Year × Treatment interactions) between factors Year (fixed: 2014, 2016), Treatment (fixed: Control (No), Low Medium, High) and Area (random: Axmouth, Beer, Lyme Regis, West Bay) for differences in the response variables (**a**) Abundance and (**b**) Weight and Width for *Cancer pagurus* and *Homarus gammarus*.

(**a**)	Species	Response variable	Model					Pairwise comparisons				
		Abundance	Source	df	SS	Pseudo-*F*	*P* (perm)	Groups	2014		2017	
									t	*P* (perm)	t	*P* (perm)
	***Cancer pagurus***											
			Year (Ye)	1	1876.1	8.0341	**0.017**	No, Low	1.2323	0.3027	0.36158	0.1237
			Treatment (Tr)	3	2120	5.6038	**0.0115**	No, Med	0.8176	0.4902	3.1717	0.1284
			Area (Ar)	3	792.6	3.7197	0.065	No, High	0.6118	0.6058	3.8006	**0.0123**
			Ye × Tr	3	2415.9	3.4078	**0.028**	Low, Med	1.289	0.2835	0.55338	0.7416
			Ye × Ar	3	700.57	0.329	0.9103	Low, High	1.5921	0.1897	2.4804	**0.0072**
			Tr × Ar	9	1134.9	0.4722	1	Med, High	0.7166	0.5409	3.4912	**0.0221**
			Residual	105	77,303							
			Total	127	86,344							
	***Homarus gammarus***											
			Year (Ye)	1	5473.5	5.3445	**0.048**	No, Low	1.158	0.3334	0.1616	0.857
			Treatment (Tr)	3	4122.2	4.711	**0.019**	No, Med	0.4825	0.6651	2.5303	0.3248
			Area (Ar)	3	520.1	1.7849	0.1235	No, High	0.7915	0.493	2.571	**0.0131**
			Ye × Tr	3	1203	3.2378	**0.0484**	Low, Med	1.6058	0.312	0.36158	0.2358
			Ye × Ar	3	650.8	1.2389	0.2389	Low, High	1.1326	0.632	2.4534	**0.016**
			Tr × Ar	9	1098.2	2.012	0.148	Med, High	1.2232	0.462	1.4899	**0.0247**
			Residual	106	37,524							
			Total	127	50,592							

(**b**)	Species	Response variable	Model					Pairwise comparisons				
								2014		2017		
		Weight	Source	df	SS	Pseudo-*F*	*P* (perm)	Groups	t	*P* (perm)	t	*P* (perm)
	***Cancer pagurus***											
			Year (Ye)	1	613.85	56.26	**0.0115**	No, Low	0.2365	0.9747	3.4297	0.2358
			Treatment (Tr)	3	9.9289	1.2492	0.3469	No, Med	1.8021	0.689	3.8006	**0.0004**
			Area (Ar)	3	45.692	1.3736	0.254	No, High	1.1042	0.3422	3.1717	**0.003**
			Ye × Tr	3	53.862	6.694	**0.0124**	Low, Med	1.8311	0.1625	0.55338	**0.0032**
			Ye × Ar	3	32.732	0.984	0.4009	Low, High	1.5187	0.2254	0.8015	**0.0273**
			Tr × Ar	9	23.844	0.23894	0.9883	Med, High	0.9453	0.4016	0.95988	0.3821
			Residual	105	1089							
			Total	127	1868.5							

		Carapace width	Source	df	SS	Pseudo-*F*	*P* (perm)					
	***Cancer pagurus***											
			Year (Ye)	1	512.83	5.287	0.0873					
			Treatment (Tr)	3	7.823	1.3734	0.2868					
			Area (Ar)	3	92.33	2.4395	0.0929					
			Ye × Tr	3	12.239	1.743	0.2378					
			Tr × Ar	3	54.324	1.928	0.2138					
			Tr × Ar	9	23.748	0.553	0.671					
			Residual	105	1165							
			Total	127	1868.5							

		Weight	Source	df	SS	Pseudo-*F*	P (perm)					
	***Homarus gammarus***											
			Year (Ye)	1	218.8	1.2983	0.2367					
			Treatment (Tr)	3	928.3	1.6231	0.323					
			Area (Ar)	3	351.2	2.3503	0.097					
			Ye × Tr	3	92.93	1.092	0.7328					
			Ye × Ar	3	12.39	1.832	0.2378					
			Tr × Ar	9	133.3	1.8949	0.152					
			Residual	105	5516.3							
			Total	127	7253.2							

		Carapace length	Source	df	SS	Pseudo-F	P (perm)					
	***Homarus gammarus***											
			Year (Ye)	1	321.98	2.903	0.1278					
			Treatment (Tr)	3	24.49	1.3734	0.2868					
			Area (Ar)	3	41.723	2.4395	0.1929					
			Ye × Tr	3	78.38	0.983	0.823					
			Ye × Ar	3	29.98	1.657	0.2378					
			Tr × Ar	9	992.46	0.553	0.7681					
			Residual	96	5764.2							
			Total	105	7253.2							

Significant main and pairwise test results are displayed in bold (*P* (perm)). Degrees of freedom (df), Sum of Squares (SS) and *F* are reported.

## Discussion

We assessed the effects of increasing pot fishing effort on multiple components of a recovering temperate reef and our results demonstrate for the first time that: (1) high densities of pot fishing can negatively affect the abundance of both sessile Reef Building taxa and target fishery species in a partially protected temperate MPA and (2) sustainable limits of pot fishing are possible and that a pot fishing intensity threshold exists. Below this threshold, the static fishery could be seen as compatible with the temperate reef ecosystem tested. This is the first time such a threshold has been demonstrated for commercial pot fishing and we have shown that, in this study, effects are likely to occur when densities of pots exceed those represented by the Medium density treatment (15–25 pots per 0.25 km^2^). On account of this evidence, we conclude that the effects of pot fishing cannot now be considered as universally benign as previously reported elsewhere^25^, but also that if managed correctly this fishery could provide a sustainable livelihood within comparable MPAs.

Both the Ross coral (*P. foliacea*) and the Neptune’s Heart sea squirt (*P. mammillata*) Reef Building taxa are previously known to be affected by bottom-towed fishing yet, to this point, are not considered to be affected by commercial pot fishing.

*P. foliacea* is a large, erect bryozoan with low recoverability, noted for being extremely slow growing^33^. At the time of the study, the seabed within the MPA was in a state of recovery, with *P. foliacea* being found very sparsely. In this study we show that if pot fishing is removed altogether, then recovery of this sensitive Reef Building taxa can be accelerated. It is therefore suggested that the presence of any pot fishing activity slows or halts the recovery of *P. foliacea.* Recovery of *P. foliacea* when exposed to pot fishing pressure is likely to be possible, but this was not picked up within the time period of this study (4 years). Certainly, there is evidence that *P. foliacea* is able to exist alongside a pot fishery, as in previous studies they have shown a recovering trend as part of the reef assemblage inside the Lyme Bay MPA since its closure, despite continued static fishery activity^34^.

*P. mammillata* is the largest solitary marine tunicate inhabiting waters of the British Isles^35^. Typically found growing on hard substratum and sediment veneers^32^, it is considered to have medium survivability to disturbance^36^. *P. mammillata,* while still in recovery, is more common within the Lyme Bay MPA^31^ and was frequently observed in the treatments of this current study. Here we demonstrate that when pot fishing density is sustained at levels equivalent to the Medium and High treatments, their numbers are reduced.

Both of these Reef Building Indicator taxa play an important role in the formation of complex biogenic reef in Lyme Bay, providing structural complexity to the seabed and acting, in part, as ecosystem engineers, offering habitats that act as nurseries, protection from predation and safe settlement opportunities for larvae of commercially important, and other, taxon^37–40^. Both *P. foliacea* and *P. mammillata* were selected as Reef Building Indicator taxa and are identified as being long lived and having low (*P. foliacea*) to medium (*P. mammillata*) recoverability after disturbance^33^.

Observed declines in the abundances of both these Reef Building Indicator taxon are likely the result of repeated hauling and deployment of pot fishing gear in addition to subsurface movements of pots related to weather and tidal movements, which, over time may have physically damaged these sensitive taxa with slower recovery rates^30,41^. Such declines under high fishing pressure could potentially prohibit temperate reef ecosystems from contributing fully to their ecosystem function. The Indicator taxa assessed here are indicative of other taxa with similar traits and life histories. It is therefore likely that other similar reef building taxa could be affected by comparative levels of pot fishing pressure.

Interestingly, although not significant our results show that the Parchment worm (*Chaetopterus variopedatus*) a tube building, tube dwelling annelid showed a declining trend with increasing pot density. This Sessile Reef Associate forms hard structures likely to be effected by pot fishing activity in a similar way to *P. foliacea* and *P. mammillata*, however, the recoverability of *C. variopedatus* is higher in comparison and recovery of this taxa in between episodes of disturbance from pots likely masks clear impacts from pot fishing. Nevertheless, it is conceivable that the number of similar benthic taxa impacted by high levels of pot fishing is likely greater than the two taxa we have shown but have not been detected in this study. We do, however, acknowledge that the majority of reef taxa assessed did not show any response to increasing pot fishing density.

Many of the ecosystem processes and services are regulated by these structure forming taxa, including supporting commercial pot fisheries^42^. Lower abundance of targeted crab and lobster in higher pot density treatments suggests that the sustained removal (by fishermen) of more individuals could be altering their abundance locally. This decline could also be associated with the observed declines in Reef Builders, known to provide important habitat for reef associated fauna; however, the effects of this interdependence are likely to be observed after a longer time period than this study was run, as no effect on reef associated fauna was noted here. The observed lower quality of brown crab in the same treatments could be linked to selective harvesting behaviours imposed by commercial fishermen, selecting for heavier (weight) crabs to retain and land as catch value is linked to landed weight. An in situ assessment of individuals is undertaken by each fisher to determine each individual crab’s muscular content, which is usually much lower if an individual is in poor health or in close proximity to a recent moult. As a consequence, more lightweight crabs are typically returned to the sea by fishers, regardless of their size, on account of having lower muscle content and subsequently worth less at market. The frequency of this selective practice, maximised in treatments of high pot density, could help explain the results seen here.

The Lyme Bay MPA was originally designated with the objective to recover rocky reef habitat by protecting all of the seabed and its epibenthic assemblages from bottom-towed fishing, and example of the Whole Site Approach^32,43^. The MPA remains partially protected and commercial pot fishing is still permitted. Based on the effects shown here, if commercial pot fishing is allowed to reach the high densities as outlined in this study then this could not only compromise the MPA objective but also demonstrate a failing in applying ecosystem-based management approaches to avoid overfishing^44,45^. We consider our conclusions to be applicable to ecologically similar ecosystems both nationally and internationally, based on the commonality of the benthic habitat and epibiota tested, and the representativity of the Indicators selected.

At a national level, over half of the UK’s MPAs have been introduced to protect reef ecosystems or features^46^. MPAs that restrict bottom-towed fishing methods could lead to an increase in pot fishing effort, already anecdotally observed within some UK MPAs^47^ and quantified in Lyme Bay^22,48^. While the effects of commercial pot fishing observed here are relatively low in comparison to the effects observed from fishing methods perceived to be more damaging, it is advised that partially protected MPAs that permit commercial pot fishing to continue and expand should therefore consider developing and incorporating adaptive management approaches in accordance with individual MPA, and national, conservation objectives and targets. There are also economic incentives for local commercial fishermen in maintaining low levels of pot fishing, which could improve efficiency and maximise economic return and quality of catch. In general, our results support the existence of low level commercial pot fishing inside MPAs, levels typically employed by the small-scale local fishermen inside the Lyme Bay MPA.

We conclude that there is an optimal level (density) of pot fishing that centers around a low-effort-high-reward strategy for both fisheries and conservation. This is an important step towards achieving well-managed partially protected MPAs and progresses our understanding regarding the ecosystem effects of different commercial fishing methods. As attitudes and behaviours change in management and the commercial fishing sector, exploitation of non-quota species without restriction is likely to continue to increase. We call on national marine managers to reconsider the conservation ambitions of partially protected MPAs in light of the effects elevated levels of pot fishing effort can cause. There are likely to be alternative commercial fishing methods allowed within partially protected MPAs that, like pot fishing, are not currently considered to be damaging to the ecosystem, but this conclusion is due to lack of assessment of impact. If these fishing methods are less restrictive in management, yet target species are high in economic value, over exploitation is perhaps inevitable. We therefore suggest applying our approach in identifying fishing intensity thresholds for such commercial fishing methods in order to achieve well-managed partially protected MPAs that can deliver benefits for both fisheries and conservation.

## Methods

### Study site

Lyme Bay is a 2460 km^2^ extent of English Channel coastline located off Devon and Dorset, South West England (Fig. 1). Lyme Bay is a ‘biodiversity hotspot’ with important submerged geological features encouraging a mosaic of habitats including sandstone, mudstone and limestone reefs and comprising of complex mixed bedrock, stony and biogenic reefs^49–51^. These habitats fall under Annex I reef habitat classification of the EU Habitats Directive^52^. Reefs and associated reef taxon were protected in 2008 by a 'Statutory Instrument' (SI), legally implemented by the United Kingdom (UK) central government's Department of Environment, Food and Rural Affairs (Defra), excluding all bottom-towed gear within a 206 km^2^ (60 nm^2^) part of Lyme Bay (Fig. 1). Within the SI boundary, static gear fishing (pots and nets), rod and line fishing and SCUBA diving to collect scallops are permitted. The SI closure forms the boundary of the Lyme Bay Marine Protected Area (MPA). The MPA has shown signs of recovery by a number of different benthic reef taxa^31,32^. Commercial pot fishing has increased within the protected Lyme Bay area since the removal of towed gear^22,48^ and so Lyme Bay was used as a test site for assessing the ecosystem effects of increasing pot densities on the recovering reef habitats.

### Experimental study

Pot densities were manipulated for four years (2014–2017), within 16 treatment units (Fig. 1), to allow for the development of fishery impacts.

Four experimental pot density treatments were introduced (1) control (no pots), (2) low pot density, (3) medium pot density and (4) high pot density. Each treatment was replicated four times and randomly interspersed within ‘Areas’ (Axmouth, Beer, Lyme Regis, West Bay) throughout the Lyme Bay MPA to account for geographical variation (Fig. 1). Each treatment unit measured 500 m × 500 m and density of pots equated to: control (no pots) = 0 pots per 0.25 km^2^, low = 5–10 pots per 0.25 km^2^, medium = 15–25 pots per 0.25 km^2^ and high = 30 pots and higher per 0.25 km^2^. These values were decided upon through consultation with local Lyme Bay Consultative Commitee fishing practitioners. Units were validated, through video surveys, that they contained homogeneous mixed ground or rocky reef substrata, between depths of 25 and 31 m. Pot densities were maintained within each unit by static gear fishermen from each port (Axmouth, Beer, Lyme Regis, West Bay). Regular commercial pot fishing trips were carried out within each unit by commercial fishermen to replicate 'normal' fishing levels, meaning two to three times per week during periods of stable weather, typically summer months, and one haul per week during periods of unsettled weather, typically winter months. Despite temporal variation in hauling activity, hauling was replicated at each timepoint within all treatments to account for variation.

The densities used in the High treatment were considered to represent maximum fishing effort per 500 m × 500 m. Assessments of pot fishing effort throughout the Devon and Severn Inshore Fishery and Conservation Authority (IFCA) district in 2008 demonstrated that 36 pots per 0.25 km^2^ was deemed to be the maximum number of pots that is viable and economical (D&S IFCA *pers comm*.). Current levels of pot fishing effort inside the Lyme Bay MPA were characterised by the Medium density. Low pot densities were also considered to replicate the pot fishing effort in some locations of the Lyme Bay MPA and were considered a level of pot fishing more similar to that of pre-closure. Controls, where pots were removed to simulate a ‘no pot fishing' treatment, were incorporated into the study as a reference point to determine baseline changes, and fishermen maintained these as no-take zones throughout the study.

**Figure 5:**
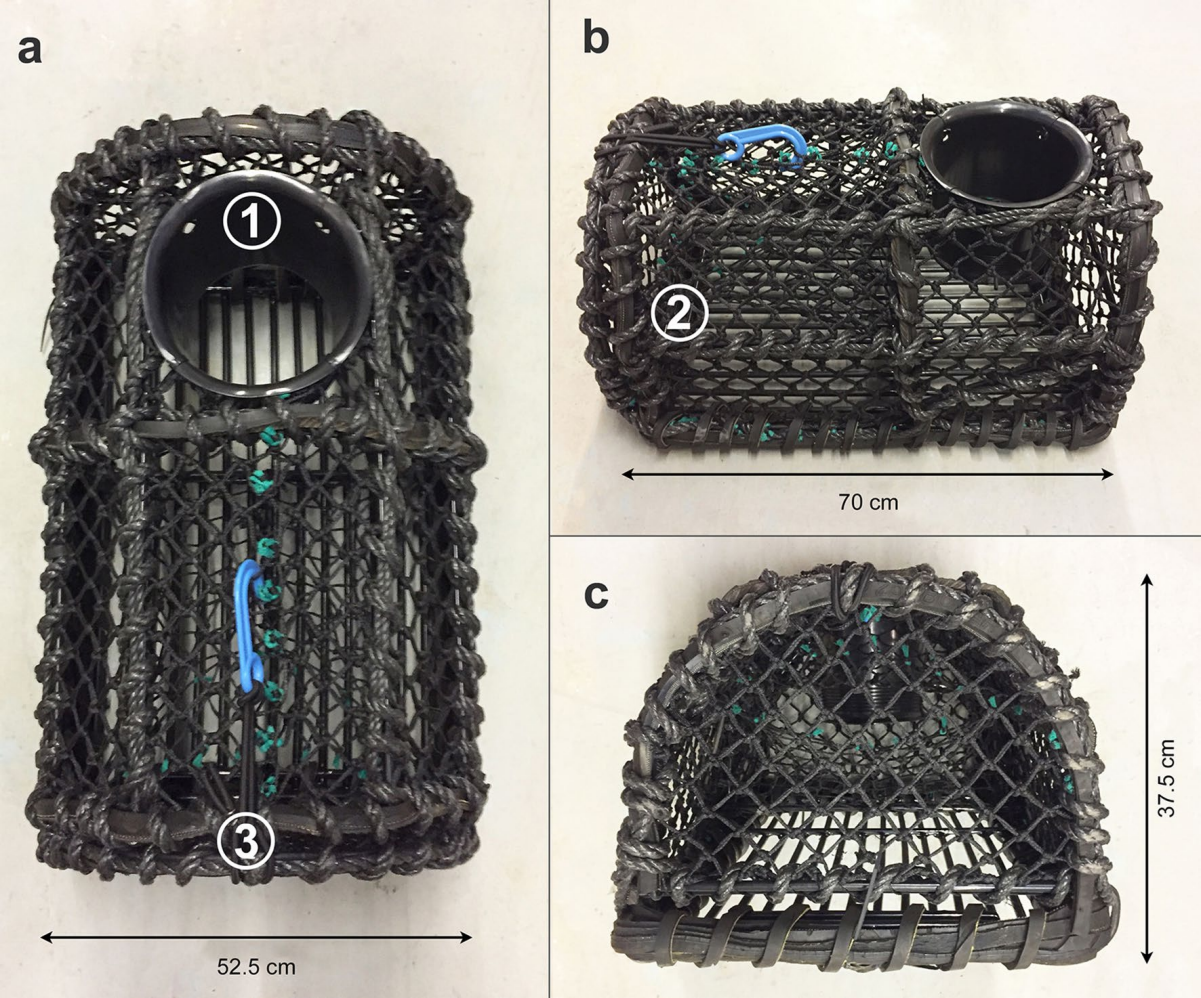
Experimental parlour pots used for quantitative pot sampling. (**a**) Planar view of singular parlour pot identifying mouth entrance (1) and release door (3), (**b**) side view of parlour pot identifying the ‘parlour’ (2), and (**c**) end view of parlour pot.

To aid pot density manipulation, experimental sets of 30 parlour pots were assigned to each port to supplement density manipulation. All pots were industry standard, measuring 70 × 52.5 × 37.5 cm. Pots had a mesh (net) size of 40 mm and each pot had a 25 cm entrance (Fig. 5). All pots were fitted with escape gaps of 84 mm wide by 46 mm high and 100 mm long, to meet Devon and Severn IFCA technical permit requirements for commercial pot fishing [Devon and Severn Inshore Fisheries and Conservation Order 2010 (S.I. 2010 No. 2212)].

Pre deployment, baseline data (2014) were compared to ensure comparability between Treatments. Treatments were environmentally, spatially and temporally replicable and started from similar ecological baselines at the start of the study (Table 1, Supplementary Figures S1, S2; Supplementary Table S2). Based on this, any observed differences between treatments after 4 years of manipulation were attributed to an effect of treatment rather than naturally occurring differences.

### Underwater video surveys

The experiment ran from 2014 to 2017 and underwater video sampling (towed and baited video, see below) was carried out annually (2014–2017) during summer months (June–August).

**Figure 6:**
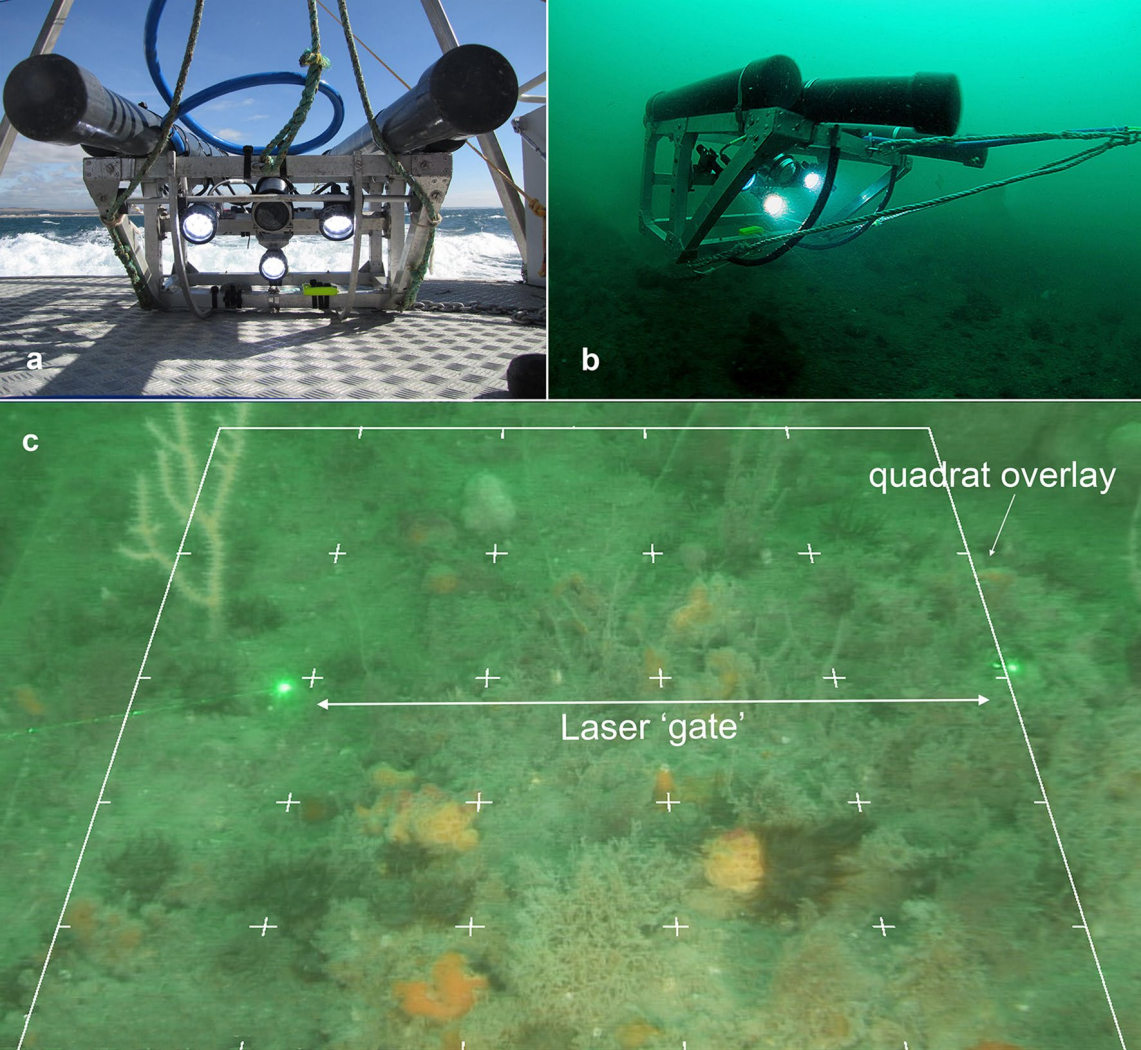
Towed video flying array and frame grab example (quadrat overlaid). (**a**,**b**) Towed video flying array; (**c**) example frame grab taken from a 50 m transect of video with digital quadrat overlaid and scaling lasers.

A towed flying array (Fig. 6) was used to capture video from transects undertaken within each of the 16 treatment units. This non-destructive and cost-effective high definition video sampling technique has been employed to quantify benthic habitats and taxa in Lyme Bay since 2008^53,54^. The array was towed behind a fishing boat (Miss Pattie, a 10 m trawler) at a speed of around 0.3 knots. The system includes a High Definition camera (Surveyor-HD-J12 colour zoom titanium, 720p), three LED lights, two green laser pointers and a mini CTD profiler. Power and signal supply were tethered to the survey vessel into a Bowtech System control unit which allows manipulation of optics. The camera is positioned at an oblique angle to the seabed to maximise field of view of the seabed. Lasers were parallel, 30 cm apart, forming a ‘gate’ that was used to measure and count epibiota (Fig. 6). As the array varies its height during sampling, this gate helps to quantify transect area (m^2^)^31,34^. Towed video data allows the confident identification and quantification of sessile and sedentary Reef Builders and Reef Associates.

Four 50 m replicate transects were carried out within each treatment unit (n = 16 per treatment = 64 for each year) at distances > 100 m from each other. Start points for each transect were predetermined using random generation of latitudinal and longitudinal coordinate seconds; however, in some instances these locations were altered in response to tidal activity and in order to avoid obstacles imposed by existing fishing gear. All benthic reef organisms were identified and enumerated using a combination of video and extracted frame grabs (10 frames per 50 m transect) from each transect and data were generated from distinct video samples. Video was used to quantify conspicuous and infrequent sessile and sedentary benthic taxa while frame grabs quantified inconspicuous and common benthic taxa. All video analyses were conducted blind with location and treatment removed to ensure no bias was introduced.

For video data, taxa that passed through the ‘gate’ were counted (full list of taxa and enumeration method see Supplementary Table S1). All taxa were identified down to the lowest taxonomic level possible. Taxon that were hard to distinguish from the video or frames were grouped by similar taxonomy or function. For example, many sponges could not be identified to species level and so were grouped as ‘branching sponges’ or ‘massive sponges’. For a full list of taxa please see Supplementary Table S1. Abundance and Taxon Richness were expressed as densities per square metre (individuals m^−2^, Taxon Richness m^−2^). Transect width was fixed (30 cm laser gate × 50 m transect length), and densities per square metre were able to be calculated using a scaling factor dependent on the position of the lasers in each frame grab (for full methods see^50^).

Frame grabs were selected every 3 s using 3Dive Frame Extractor. If frame grab images were not in focus, did not show > 50% hard substratum (cobble, boulders or rock), excluded lasers, overlapped with previous frames or if the benthos was obscured by larger taxa then they were removed before 10 were randomly selected^34,50^. A digital quadrat was overlaid on each frame grab. The quadrat overlay comprised 16 equidistant dots overlaid on to each frame. The percentage cover of encrusting, colonial taxa were quantified by dividing the number of dots that covered each taxon by the total number of dots^31,34^. Mixed low-lying hydroid and algae communities that were under 1 cm in height were recorded as ‘Turf’ and also quantified as a percentage cover.

**Figure 7:**
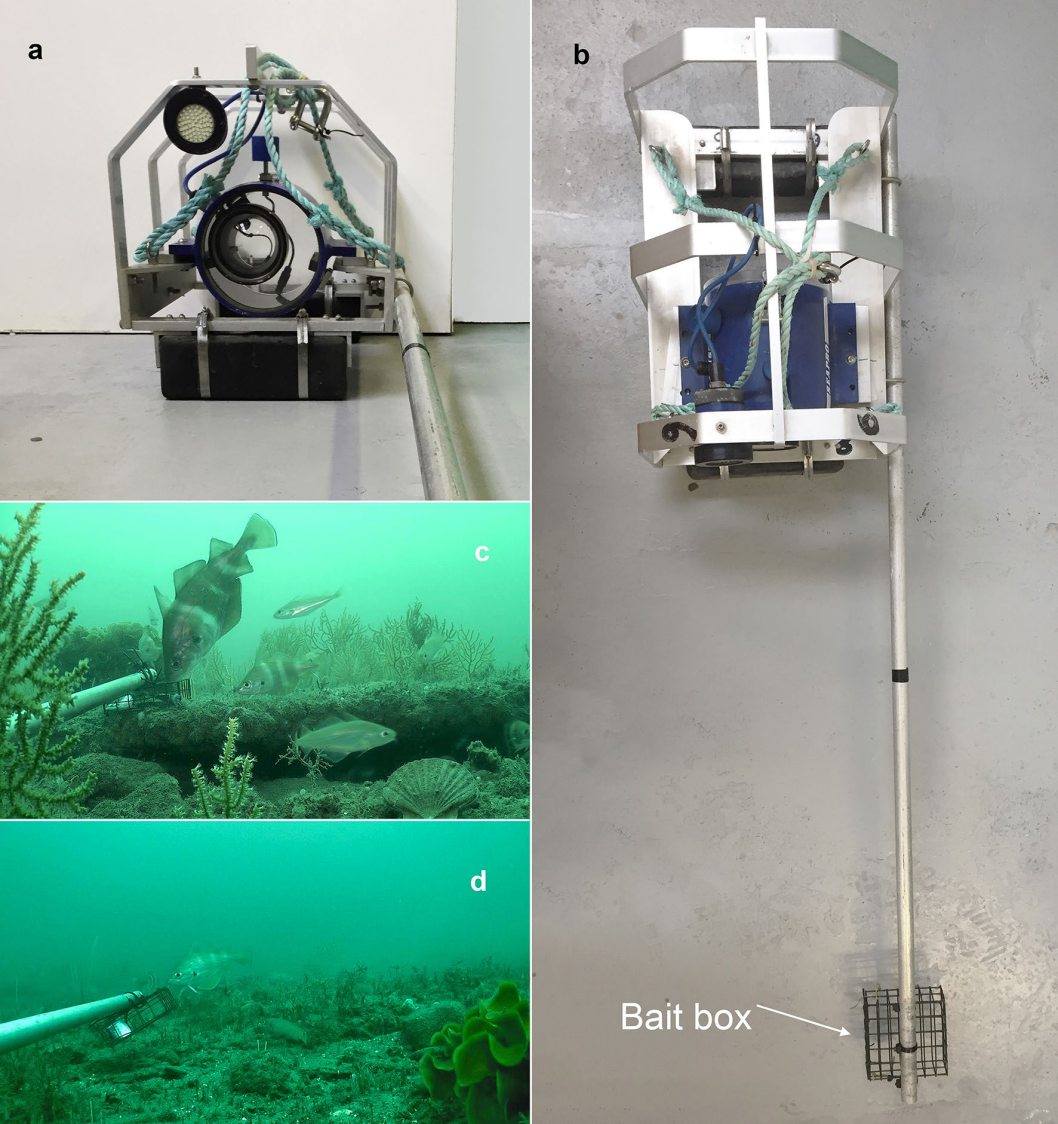
Baited remote underwater video (BRUV) system and sample video stills. (**a**) Front view of BRUV system showing weight, camera housing and light, (**b**) planar view of system with bait arm and bait box attached, (**c**,**d**) sample stills taken from a video recording showing bait box in foreground with (**c**) pouting (*Trisopterus luscus*) and Poor Cod (*Trisopterus minutus*) and (**d**) pouting (*Trisopterus luscus*).

Mobile organisms were sampled using a Baited Remote Underwater Video (BRUV) approach^31,34,50^. BRUV systems were deployed from two local fishing vessels based out of the port of Lyme Regis. Each BRUV system was equipped with a full HD Video Camera housed within Seapro Subsea video camera modules, with a single diffused LED light. Cameras auto focused through a Wideangle Seapro Optolite Port lens which had a concave inner surface and flat front, providing a wide field of view (Fig. 7). This allowed a sharp focus from a few mm in front of the port to infinity, providing suitable optical flexibility for identification. A pole held a wire mesh bait box 1 m away from the cameras and contained 100 g of mackerel (*Scomber scombrus,* replenished for each replicate) as an attractant. Each rig had two 15 kg lead weights attached to their base to provide stability against tidal currents.

Within each treatment unit, at two randomly predetermined sites, sets of three BRUVs were deployed haphazardly a minimum of 50 m apart (per site) from each other for 40 min (n = 6 per unit = 24 per treatment = 96 per year). BRUVs were given 5 min to allow disturbed sediments to settle and to allow an olfactory trail to be established^55^. Site depths and sea surface temperatures varied from 25.4 to 28 m and 14 to 18.4 °C.

From each 30 min sample, data were extracted using normal speed playback, during which all macro-mobile taxa entering the field of view were recorded and identified to the lowest taxonomic level possible. Goby taxa (Gobidae) were grouped as ‘Grouped gobies’ due to being hard to identify consistently, while spider crab genera *Inachus* and *Macropodia* were recorded as *Inachus* spp. and *Macropodia* spp. due to being taxonomically similar or hard to distinguish from each other (Supplementary Table S1). The maximum number of individuals of the same taxon appearing on screen per one minute slices of video was recorded. The highest value recorded from 30 individual counts was then used as a measure of relative abundance referred to as MaxN^40,56–59^. MaxN ensures repeat counts of individuals re- entering the field of view are avoided^56,57,60,61^. Video analysis was undertaken blind with no indication of video location, site or treatment and data were generated from distinct BRUV samples.

123 taxon, or groups of taxonomically similar taxa, were recorded through underwater video surveys (Supplementary Table S1). Prior to analysis, taxon were grouped based on their ecological function, determined using expert knowledge and previous research^33^. All identifiable taxa were enumerated once on either towed or baited video survey and included in one functional group only. Functional Groups: ‘Reef Builders’ = Individual or colonial sessile reef taxon that form large, erect structures on hard substratum, enumerated using towed video (18 taxa); ‘Reef Associates’ = Taxa living and feeding on or near reef^62^. Reef Associates were subdivided by their motility into ‘Sessile and Sedentary Reef Associates’ (64 taxa), enumerated using towed video, and ‘Mobile Reef Associates’ (43 taxa), enumerated using baited video (Supplementary Table S1). Encrusting algae were removed altogether.

Abundance of nine preselected ‘Indicator taxa’ were analysed separately. Indicator taxa were preselected from an adapted list of taxa used to inform the Lyme Bay long term monitoring project, originally selected for their varying biological characteristics relating to life history that are relevant to recoverability from disturbance^32,33,36^. Here, we selected Indicator taxa that were first identified as Indicators from the predetermined list^33^, and, secondly, Indicators that also represented each functional Group (Reef Builders, Reef Associates (Sessile and Sedentary, Mobile)). Originally the anemone *Aiptasia mutabilis* was identified to be an Indicator taxa; however, a number of morphologically similar anemones were identified during the study, so these taxa were grouped as the Sessile and Sedentary Reef Associate Indicator ‘Grouped large anemones’ (Supplementary Table S1).

### Pot fishing survey

Commercial fishery sampling was carried out using pot fishing surveys undertaken seasonally [Spring (March), Summer (June), Autumn (September) and Winter (December/January)] in the years 2014, 2015 and 2016 only of this study. Year 4 pot fishing surveys were not undertaken due to funding constraints deliver this element of the program.

Pot fishing surveys were undertaken within each treatment unit to collect Abundance data on commercially targeted species and bycatch. To account for seasonal variation, sampling periods occurred every three months: Spring (March), Summer (June), Autumn (September) and Winter (December/January) in the years 2014, 2015 and 2016. In each sampling period 30 experimental pots (see above for pot specifications, Fig. 5) divided into six strings of five pots, were baited and haphazardly deployed throughout each treatment unit once (16 units in total, see Fig. 1). In order to representatively sample the entire population, escape gaps were closed for sampling, with dispensation from Devon and Severn IFCA. Frozen ‘Scad’ (*Trachurus trachurus*) was used for bait due to its suitability, low economic cost and annual availability. Pots were left to ‘soak’ for a 24 h period and then hauled. All organisms were identified and counted. For brown crab (*Cancer pagurus*) and European lobster (*Homarus gammarus*) the following biometrics were also recorded: Carapace Width (CW) (brown crab only) and Carapace Length (CL) (European lobster only)) using 200 mm digital calipers. Wet weight using 10 g–40 kg digital hanging scales, sex, cheliped status (intact or number missing), and signs of ovigery were also recorded. After sampling, all organisms were returned within the treatment unit from which they were collected. All methods were carried out in accordance with relevant guidelines and regulations of the University of Plymouth for the handling of live invertebrates. All experimental protocols were approved by the University of Plymouth.

### Data analysis

Data were formally compared between year and treatment using data from the start (2014) and the end of the study; after 4 years for underwater video survey data (2017) and after 3 years for pot fishing survey data (*C. pagurus* and *H. gammarus* only) (2016). These were the last years each survey type was carried out.

Permutational Multivariate Analysis of Variance (PERMANOVA+ using PRIMER v7 software package)^63^ was used to compare data between response variables: Reef Builders, Reef Associates (Sessile and Sedentary, Mobile) = Abundance, Taxon Richness; Commercial fishery = Abundance, Carapace Width (CW) and Weight). The response variables were tested between: Year (fixed: 2014, 2017), Area (random: Axmouth, Beer, Lyme Regis, West Bay) and Treatment (fixed: Control, Low, Medium, High) using the following replication: Reef Builders and Sessile and Sedentary reef Associates replicates = average (individuals per m^−2^) of combined data generated from both the 50 m video and the 10 selected frame grabs of each transect [one transect = one replicate (n = 16 per Treatment)]; Mobile Reef Associates = average (MaxN) for set of three (per site) BRUV deployments [Three BRUVs = one replicate, (n = 8 per Treatment)]; Commercial fishery = average of 5 pots (string) [one string = one replicate (n = 24 per Treatment)] (Supplementary Table S1). Each term in the analysis used 9999 unrestricted permutations of raw data and Type I SS^64^. PERMANOVA is robust to datasets with many zeros, makes no assumptions about underlying data distributions and allows the testing of interactions in complex multifactorial designs^65^. Commercial fishery data were analysed without prior transformation while all other data were first 4th root transformed. All analyses were based on Euclidean distance similarity matrices. A priori *P* values of < 0.05 were used to determine significance and significant differences between Treatments were explored further using pairwise tests.

## Supplementary Information

Supplementary Information

## Data Availability

A complete list of taxa is provided in Supplementary Table S1. The datasets generated during and/or analysed during the current study will be archived in the Marine Biological Association repository (DASSH, The Archive for Marine Species and Habitats Data), and made available via the MEDIN (Marine Environmental Data and Information Network) portal (https://portal.medin.org.uk/portal/start.php). Please contact the corresponding author via adam.rees@plymouth.ac.uk for further information.

## References

[1] Hall-Spencer JM, Moore PG (2000). Scallop dredging has profound, long-term impacts on maerl habitats. ICES J. Mar. Sci..

[2] Eigaard OR (2017). The footprint of bottom trawling in European waters: Distribution, intensity, and seabed integrity. ICES J. Mar. Sci..

[3] Auster PJ (1996). The impacts of mobile fishing gear on seafloor habitats in the gulf of maine (Northwest Atlantic): Implications for conservation of fish populations. Rev. Fish. Sci..

[4] Gell FR, Roberts CM (2003). Benefits beyond boundaries: The fishery effects of marine reserves. Trends Ecol. Evol..

[5] Roberts CM (2017). Marine reserves canmitigate and promote adaptation to climate change. Proc. Natl. Acad. Sci. USA.

[6] Sciberras M, Jenkins SR, Kaiser MJ, Hawkins SJ, Pullin AS (2013). Evaluating the biological effectiveness of fully and partially protected marine areas. Environ. Evid..

[7] Afonso P, Schmiing M, Diogo H, Serra R (2015). With various conservation objectives and targets. ICES J. Mar. Sci..

[8] Schmiing M, Diogo H, Santos RS, Afonso P (2015). Marine conservation of multispecies and multi-use areas with various conservation objectives and targets. ICES J. Mar. Sci..

[9] Giakoumi S (2017). Ecological effects of full and partial protection in the crowded Mediterranean Sea: A regional meta-analysis. Sci. Rep..

[10] Zupan M (2018). Marine partially protected areas: Drivers of ecological effectiveness. Front. Ecol. Environ..

[11] Halpern BS (2003). The impact of marine reserves: Do reserves work and does reserve size matter. Ecol. Appl..

[12] Pikitch EK (2004). Ecosystem-based fishery management. Science (80–).

[13] Claudet J (2008). Marine reserves: Size and age do matter. Ecol. Lett..

[14] Lester SE (2009). Biological effects within no-take marine reserves: A global synthesis. Mar. Ecol. Prog. Ser..

[15] Fraschetti S, Guarnieri G, Bevilacqua S, Terlizzi A, Boero F (2013). Protection enhances community and habitat stability: Evidence from a Mediterranean marine protected area. PLoS One.

[16] Kerwath SE, Winker H, Götz A, Attwood CG (2013). Marine protected area improves yield without disadvantaging fishers. Nat. Commun..

[17] Edgar GJ (2014). Global conservation outcomes depend on marine protected areas with five key features. Nature.

[18] Hiddink JG (2017). Global analysis of depletion and recovery of seabed biota after bottom trawling disturbance. Proc. Natl. Acad. Sci. USA.

[19] Lombard AT (2019). Key challenges in advancing an ecosystem-based approach to marine spatial planning under economic growth imperatives. Front. Mar. Sci..

[20] Trochta JT (2018). Ecosystem-based fisheries management: Perception on definitions, implementations, and aspirations. PLoS One.

[21] EEA. *Marine Protected Areas in Europe’s Seas. An Overview and Perspectives for the Future.* (2015). 10.2800/99473.

[22] Mangi SC, Rodwell LD, Hattam C (2011). Assessing the impacts of establishing MPAs on fishermen and fish merchants: The case of Lyme Bay, UK. Ambio.

[23] Luisetti T (2011). Coastal and marine ecosystem services valuation for policy and management: Managed realignment case studies in England. Ocean Coast. Manag..

[24] Molfese C, Beare D, Hall-Spencer JM (2014). Overfishing and the replacement of demersal finfish by shellfish: An example from the english channel. PLoS One.

[25] Eno NC (2001). Effects of crustacean traps on benthic fauna. ICES J. Mar. Sci..

[26] Coleman RA, Hoskin MG, von Carlshausen E, Davis CM (2013). Using a no-take zone to assess the impacts of fishing: Sessile epifauna appear insensitive to environmental disturbances from commercial potting. J. Exp. Mar. Bio. Ecol..

[27] Lewis CF, Slade SL, Maxwell KE, Matthews TR (2009). Lobster trap impact on coral reefs: Effects of wind-driven trap movement. New Zeal. J. Mar. Freshw. Res..

[28] Micheli F, De Leo G, Butner C, Martone RG, Shester G (2014). A risk-based framework for assessing the cumulative impact of multiple fisheries. Biol. Conserv..

[29] Stephenson F, Mill AC, Scott CL, Polunin NVC, Fitzsimmons C (2017). Experimental potting impacts on common UK reef habitats in areas of high and low fishing pressure. ICES J. Mar. Sci..

[30] Sinclair M, Valdimarsson G (2014). Responsible fisheries in the marine ecosystem. Fish. Res..

[31] Sheehan EV, Stevens TF, Gall SC, Cousens SL, Attrill MJ (2013). Recovery of a temperate reef assemblage in a marine protected area following the exclusion of towed demersal fishing. PLoS One.

[32] Sheehan EV (2013). Drawing lines at the sand: Evidence for functional vs visual reef boundaries in temperate Marine Protected Areas. Mar. Pollut. Bull..

[33] Jackson, E. L., Langmead, O., Barnes, M., Tyler-Walters, H. & Hiscock, K. *Lyme Bay—A Case Study: Measuring Recovery of Benthic Species, Assessing Potential Spill-Over Effects and Socio-economic Changes*. (2008).

[34] Stevens TF, Sheehan EV, Gall SC, Fowell SC, Attrill MJ (2014). Monitoring benthic biodiversity restoration in Lyme Bay marine protected area: Design, sampling and analysis. Mar. Policy.

[35] Picton BE, Morrow CC (2016). Encyclopedia of Marine Life of Britain and Ireland.

[36] Langmead. *Lyme Bay—A Case Study: Measuring Recovery of Benthic Species, Assessing Potential Spill-Over Effects and Socio-Economic Changes*. 44 (2012).

[37] Bradshaw C, Collins P, Brand AR (2003). To what extent does upright sessile epifauna affect benthic biodiversity and community composition?. Mar. Biol..

[38] Cocito S, Ferdeghini F, Sgorbini S (1998). Pentapora fascialis (Pallas) [Cheilostomata: Ascophora] colonization of one sublittoral rocky site after sea-storm in the northwestern mediterranean. Hydrobiologia.

[39] Eggleston D, Lipcius R, Miller D, Coba-Cetina L (1990). Shelter scaling regulates survival of juvenile Caribbean spiny lobster *Panulirus argus*. Mar. Ecol. Prog. Ser..

[40] Pirtle JL, Eckert GL, Stoner AW (2012). Habitat structure influences the survival and predator-prey interactions of early juvenile red king crab *Paralithodes camtschaticus*. Mar. Ecol. Prog. Ser..

[41] Gall SC (2020). The impact of potting for crustaceans on temperate rocky reef habitats: Implications for management. Mar. Environ. Res..

[42] Lambert GI, Jennings S, Kaiser MJ, Hinz H, Hiddink JG (2011). Quantification and prediction of the impact of fishing on epifaunal communities. Mar. Ecol. Prog. Ser..

[43] Soldant J, Mullier T, Elliott T, Sheehan EV, Humphreys J, Clark RWE (2020). Managing marine protected areas in Europe: Moving from ‘feature-based’ to ’whole-site; management of sites. Marine Protected Areas: Science, Policy and Management.

[44] Staples D, Funge-Smith S (2009). Ecosystem Approach to Fisheries and Aquaculture: Implementing the FAO Code of Conduct for Responsible Fisheries.

[45] Garcia SM, Rice J, Charles A (2015). Bridging fisheries management and biodiversity conservation norms: Potential and challenges ofbalancing harvest in ecosystem- based frameworks. Nature.

[46] DEFRA. *Marine Protected Areas Network Report 2012–2018*. (2018).

[47] Burke C (2015). Ireland’s need for inshore local management. Fish. News.

[48] Rees, S. E. *et al. An evaluation Framework to Determine the Impact of the Lyme Bay Fisheries and Conservation Reserve and the Activities of the Lyme Bay Consultative Committee on Ecosystem Services and Human Wellbeing Final Report To the October 2016*. (2016).

[49] Cork, M., McNulty, S. & Gaches, P. *Site Selection Report for Inshore Marine SACs Project. Poole Bay to Lyme Bay*. *Report No. 9S0282/SSR/PooleLymeBay/01* (2008).

[50] Attrill, M. J. *et al. Lyme Bay—A Case Study: Measuring Recovery of Benthic Species, Assessing Potential Spill-Over Effects and Socio-economic chaNges*. (2012).

[51] Ross, R. *South Devon Reef Video Baseline Surveys for the Prawle Point to Plymouth Sound & Eddystone cSAC and Surrounding Areas As commissioned by Natural England South Devon Reef Video Baseline Surveys for the Prawle Point to Plymouth Sound & Eddystone cSAC and Su*. (2016)10.13140/2.1.2313.1205.

[52] Vanstaen, K. & Eggleton, J. *Mapping Annex 1 Reef Habitat Present in Specific areas Within the Lyme Bay and Torbay cSAC*. (2011).

[53] Sheehan EV, Stevens TF, Attrill MJ, Ropert-Coudert Y (2010). A quantitative, non-destructive methodology for habitat characterisation and benthic monitoring at offshore renewable energy developments. PLoS ONE.

[54] Sheehan EV, Vaz S, Pettifer E, Foster NL, Nancollas SJ, Cousens S, Holmes L, Facq J-V, Germain G, Attrill MJ, Reynolds J (2016). An experimental comparison of three towed underwater video systems using species metrics, benthic impact and performance. Methods in Ecology and Evolution.

[55] Bicknell AWJ, Sheehan EV, Godley BJ, Doherty PD, Witt MJ (2019). Assessing the impact of introduced infrastructure at sea with cameras: A case study for spatial scale, time and statistical power. Mar. Environ. Res..

[56] Priede IG, Bagley PM, Smith A, Creasey S, Merrett NR (1994). Scavenging deep demersal fishes of the porcupine seabight, North-East Atlantic: Observations by baited camera, trap and trawl. Nat. Hist..

[57] Watson DL, Harvey ES, Anderson MJ, Kendrick GA (2005). A comparison of temperate reef fish assemblages recorded by three underwater stereo-video techniques. Mar. Biol..

[58] Cappo M, Harvey E, Shortis M (2006). Counting and measuring fish with baited video techniques—an overview. Aust. Soc. Fish Biol..

[59] Elliott SAM, Turrell WR, Heath MR, Bailey DM (2017). Juvenile gadoid habitat and ontogenetic shift observations using stereo-video baited cameras. Mar. Ecol. Prog. Ser..

[60] McLean DL, Harvey ES, Fairclough DV, Newman SJ (2010). Large decline in the abundance of a targeted tropical lethrinid in areas open and closed to fishing. Mar. Ecol. Prog. Ser..

[61] Harvey ES (2012). Comparison of the relative efficiencies of stereo-BRUVs and traps for sampling tropical continental shelf demersal fishes. Fish. Res..

[62] Maragos, J. E. *Marine and Coastal Biodiversity in the Tropical Island Pacific Region*. (East-West Center, 1995).

[63] Clarke KR, Warwick RM (2001). Change in Marine Communities: An Approach to Statistical Analysis and Interpretation.

[64] Taylor P, Anderson M, Ter Braak C (2006). J. Stat. Comput. Permut. Tests Multi-Factor. Anal. Variance.

[65] Anderson MJ (2001). A new method for non-parametric multivariate analysis of variance MARTI. Austral Ecol..

