# Supplementary material for: Optimal fishing effort benefits fisheries and conservation
Source: Sci Rep. 2021 Feb 15;11:3784. doi: 10.1038/s41598-021-82847-4 (PMC7884685; doi:10.1038/s41598-021-82847-4)
Supplement: Supplementary file 1 — Supplementary Information [file 41598_2021_82847_MOESM1_ESM.pdf]

Optimal fishing effort benefits fisheries and conservation

Adam Rees[1]*, Emma V Sheehan[1]* and Martin J Attrill[1]*

[1]University of Plymouth, School of Biological and Marine Sciences

Drake Circus, Plymouth, PL4 8AA, UK

Supplementary Table 1. All taxa quantified in underwater video surveys. Each taxon (grouped if taxonomically similar) has data collection method, sample used for analysis (BRUV = Baited Remote Underwater Video), how Abundance was enumerated, and unit Abundance was expressed in. Taxon categorised as Indicator taxa are identified using ticks. Functional Group code (RB = Reef Builders; SS = Sessile and Sedentary Reef Associates; M = Mobile Reef Associated taxa) is also displayed.

Taxon	Common name	Data collection method(s)	Sample(s) used in analysis	Enumeration method	Unit	Predetermined Indicators (Jackson et al. 2008) (bold = analysed)	Group (RB = Reef Builders; SS = Sessile and Sedentary Reef Associates; M = Mobile Reef Associates)
Acromegalomma vesiculosum	A fanworm	Towed video	10 frame grabs	Count	Individuals m^{-2}	-	SS
Actinothoe sphyrodeta	Sandalled anemone	Towed video	10 frame grabs	Count	Individuals m^{-2}	-	SS
Aequipecten opercularis	Queen scallop	Towed video	50m transect video	Count	Individuals m^{-2}	-	SS
Alcyonidium diaphanum	Sea chervil	Towed video	10 frame grabs	Count	Individuals m^{-2}	-	RB
Alcyonium digitatum	Dead man's fingers	Towed video	50m transect video	Count	Individuals m^{-2}	-	RB
Anseropoda placenta	Goose foot starfish	Towed video	50m transect video	Count	Individuals m^{-2}	-	SS
Aphrodita aculeata	Sea mouse	Towed video	10 frame grabs	Count	Individuals m^{-2}	-	SS
Aslia lefevrei	Brown sea cucumber	Towed video	10 frame grabs	Count	Individuals m^{-2}	-	SS
Asterias rubens	Common starfish	Towed video	50m transect video	Count	Individuals m^{-2}	✓	SS
Astropecten irregularis	Sand sea star	Towed video	50m transect video	Count	Individuals m^{-2}	-	SS
Bispira volutacornis	Twin fan worm	Towed video	10 frame grabs	Count	Individuals m^{-2}	-	SS
Blennius ocellaris	Butterfly blenny	Towed video	50m transect video	Count	Individuals m^{-2}	-	SS
Buccinum undatum	Common whelk	Towed video	50m transect video	Count	Individuals m^{-2}	-	SS
Bugulina spp.	Grouped Bugulina bryozoans	Towed video	10 frame grabs	Count	Individuals m^{-2}	-	SS
Callionymus lyra	Common dragonet	Baited video	30 minute BRUV sample	Count	MaxN	-	M
Calliostoma zizyphinum	Painted top shell	Towed video	10 frame grabs	Count	Individuals m^{-2}	-	SS
Cancer pagurus	Brown crab	Baited video	30 minute BRUV sample	Count	MaxN	-	M
Caryophyllia (Caryophyllia) smithii	Devonshire cup coral	Towed video	10 frame grabs	Count	Individuals m^{-2}	-	SS
Cellaria fistulosa	An erect bryozoan	Towed video	10 frame grabs	Count	Individuals m^{-2}	-	SS
Cellepora pumicosa	An encrusting bryozoan	Towed video	10 frame grabs	Count	Individuals m^{-2}	-	SS
Centrolabrus exoletus	Rock cook wrasse	Baited video	30 minute BRUV sample	Count	MaxN	-	M
Cereus pedunculatus	Daisy anemone	Towed video	10 frame grabs	Count	Individuals m^{-2}	-	SS
Chaetopterus variopedatus	Parchment worm	Towed video	10 frame grabs	Count	Individuals m^{-2}	✓	SS
Chelidonichthys cuculus	Red gurnard	Baited video	30 minute BRUV sample	Count	MaxN	-	M
Chelidonichthys lastoviza	Streaked gurnard	Towed video	50m transect video	Count	Individuals m^{-2}	-	M
Chelidonichthys lucerna	Tub gurnard	Baited video	30 minute BRUV sample	Count	MaxN	-	M
Ciocalypta penicillus	A pencil sponge	Towed video	10 frame grabs	Count	Individuals m^{-2}	-	RB
Ciona intestinalis	Transparent sea squirt	Towed video	10 frame grabs	Count	Individuals m^{-2}	-	SS
Clathrina coriacea	White lace sponge	Towed video	10 frame grabs	Count	Individuals m^{-2}	-	SS
Conger conger	Conger eel	Baited video	30 minute BRUV sample	Count	MaxN	-	M
Corella parallelogramma	Gas mantle ascidian	Towed video	10 frame grabs	Count	Individuals m^{-2}	-	SS
Corynactis viridis	Jewel anemone	Towed video	10 frame grabs	Count	Individuals m^{-2}	-	SS
Ctenolabrus rupestris	Goldsinny wrasse	Baited video	30 minute BRUV sample	Count	MaxN	-	M
Diazona violacea	Football sea squirt	Towed video	10 frame grabs	Count	Individuals m^{-2}	-	SS
Dicentrarchus labrax	Bass	Baited video	30 minute BRUV sample	Count	MaxN	-	M
Dysidea fragilis	Goosebump sponge	Towed video	10 frame grabs	Count	Individuals m^{-2}	-	SS
Epitonium clathrus	Common wentletrap	Towed video	10 frame grabs	Count	Individuals m^{-2}	-	SS
Epizoanthus couchii	Colonial sea anemone	Towed video	10 frame grabs	Count	Individuals m^{-2}	-	SS
Eunicella verrucosa	Pink sea fan	Towed video	50m transect video	Count	Individuals m^{-2}	✓	RB
Eutrigla gurnardus	Grey gurnard	Baited video	30 minute BRUV sample	Count	MaxN	-	M
Filogran implexa	Coral worm	Towed video	10 frame grabs	Count	Individuals m^{-2}	-	SS
Flustra foliacea	Hornwrack	Towed video	10 frame grabs	Count	Individuals m^{-2}	-	RB
Gadus morhua	Cod	Baited video	30 minute BRUV sample	Count	MaxN	-	M
Gaidropsarus spp.	Grouped rocklings	Baited video	30 minute BRUV sample	Count	MaxN	-	M
Goneplax rhomboides	Angular crab	Towed video	50m transect video	Count	Individuals m^{-2}	-	SS
Grantia compressa	Purse Sponge	Towed video	10 frame grabs	Count	Individuals m^{-2}	-	SS
Grouped anemones	Grouped anemones	Towed video	10 frame grabs	Count	Individuals m^{-2}	-	SS
Grouped branching sponges	Grouped branching sponges	Towed video	10 frame grabs	Count	Individuals m^{-2}	-	RB
Grouped gobies	Grouped goby taxa	Baited video	30 minute BRUV sample	Count	MaxN	-	M
Grouped hydroids	Grouped hydroids	Towed video	10 frame grabs	Count	Individuals m^{-2}	-	RB
Grouped large anemones	Grouped large anemones (*Aiptasia mutabilis* + Cerianthid spp. + *Mesacmaea mitchellii*)	Towed video	10 frame grabs	Count	Individuals m^{-2}	✓	SS
Grouped massive sponges	Grouped massive sponges	Towed video	10 frame grabs	Count	Individuals m^{-2}	-	RB
Grouped Sagartiidae anemones	Grouped Sagartiidae anemones	Towed video	10 frame grabs	Count	Individuals m^{-2}	-	SS
Grouped small orange ascidians	Grouped baked bean ascidians	Towed video	10 frame grabs	Count	Individuals m^{-2}	-	SS
Grouped Ascidiidae	Grouped Ascidiidae (*Ascidiella aspersa + Ascidia conchilega + Ascidia mentula +Ascidia virginea*)	Towed video	10 frame grabs	Count	Individuals m^{-2}	-	SS
Grouped Suberitidae sponges	Grouped Suberitidae sponges	Towed video	10 frame grabs	Count	Individuals m^{-2}	-	RB
Hemimycale columella	Crater sponge	Towed video	10 frame grabs	Count	Individuals m^{-2}	-	SS
Homarus gammarus	European lobster	Baited video	30 minute BRUV sample	Count	MaxN	-	M
Hyas coarctatus	Toad crab	Towed video	30 minute BRUV sample	Count	MaxN	-	M
Inachus spp.	Grouped scorpion spider crabs	Baited video	30 minute BRUV sample	Count	MaxN	-	M
Janolus cristatus	A nudibranch	Towed video	10 frame grabs	Count	Individuals m^{-2}	-	SS
Labrus bergylta	Ballan wrasse	Baited video	30 minute BRUV sample	Count	MaxN	✓	M
Labrus mixtus	Cuckoo wrasse	Baited video	30 minute BRUV sample	Count	MaxN	-	M
Lanice conchilega	Sand mason	Towed video	10 frame grabs	Count	Individuals m^{-2}	-	SS
Limanda limanda	Dab	Baited video	30 minute BRUV sample	Count	MaxN	-	M
Lipophrys pholis	Shanny	Towed video	50m transect video	Count	Individuals m^{-2}	-	SS
Luidia ciliaris	Seven armed starfish	Towed video	50m transect video	Count	Individuals m^{-2}	-	SS
Macropodia spp.	Grouped long-legged spider crabs	Towed video	30 minute BRUV sample	Count	MaxN	-	M
Maja squinado	Common spider crab	Baited video	30 minute BRUV sample	Count	MaxN	-	M
Merlangius merlangus	Whiting	Baited video	30 minute BRUV sample	Count	MaxN	-	M
Metacallophyllis laciniata	Red algae	Towed video	10 frame grabs	Count	Individuals m^{-2}	-	SS
Metridium senile	Plumose anemone	Towed video	10 frame grabs	Count	Individuals m^{-2}	-	SS
Molgula manhattensis	Sea grapes	Towed video	10 frame grabs	Count	Individuals m^{-2}	-	SS
Molva molva	Common ling	Baited video	30 minute BRUV sample	Count	MaxN	-	M
Mullus surmuletus	Striped red mullet	Baited video	30 minute BRUV sample	Count	MaxN	-	M
Myxicola infundibulum	Sabellid fanworm	Towed video	10 frame grabs	Count	Individuals m^{-2}	-	SS
Myxilla fimbriata	Smooth orange encrusting sponge	Towed video	10 frame grabs	Count	Individuals m^{-2}	-	SS

Necora puber	Velvet swimming crab	Baited video	30 minute BRUV sample	Count	MaxN	✓	M
Nemertesia antennina	Sea beard	Towed video	10 frame grabs	Count	Individuals m^{-2}	-	RB
Nemertesia ramosa	Eurybathic hydrozoan	Towed video	10 frame grabs	Count	Individuals m^{-2}	-	RB
Neopentadactyla mixta	Gravel sea cucumber	Towed video	10 frame grabs	Count	Individuals m^{-2}	-	SS
Ocnus planci	Small sea cucumber	Towed video	10 frame grabs	Count	Individuals m^{-2}	-	SS
Ophiocomina nigra	Black brittlestar	Towed video	50m transect video	Count	Individuals m^{-2}	-	SS
Ophiothrix fragilis	Common brittlestar	Towed video	50m transect video	Count	Individuals m^{-2}	-	SS
Ophiura ophiura	Serpent star	Towed video	50m transect video	Count	Individuals m^{-2}	-	SS
Pagurus spp.	Grouped hermit crabs	Towed video	50m transect video	Count	Individuals m^{-2}	-	SS
Parablennius gattorugine	Tompot blenny	Towed video	50m transect video	Count	Individuals m^{-2}	-	SS
Pecten maximus	King scallop	Towed video	50m transect video	Count	Individuals m^{-2}	-	SS
Pentapora foliacea	Ross coral	Towed video	50m transect video	Count	Individuals m^{-2}	✓	RB
Phallusia mammillata	Neptune's Heart sea squirt	Towed video	50m transect video	Count	Individuals m^{-2}	✓	RB
Pholas dactylus	Common Piddock	Towed video	10 frame grabs	Count	Individuals m^{-2}	-	SS
Pholis gunnellus	Rock gunnel	Baited video	30 minute BRUV sample	Count	MaxN	-	M
Pleuronectes platessa	European plaice	Baited video	30 minute BRUV sample	Count	MaxN	-	M
Pollachius pollachius	Pollack	Baited video	30 minute BRUV sample	Count	MaxN	-	M
Polymastiaboletiformis	A cushion sponge	Towed video	10 frame grabs	Count	Individuals m^{-2}	-	RB
Polymastia penicillus	Chimney sponge	Towed video	10 frame grabs	Count	Individuals m^{-2}	-	RB
Pyura spp.	Grouped Pyura taxon	Towed video	10 frame grabs	Count	Individuals m^{-2}	-	SS
Raja clavata	Thornback ray	Baited video	30 minute BRUV sample	Count	MaxN	-	M
Sabella pavonina	Peacock worm	Towed video	10 frame grabs	Count	Individuals m^{-2}	-	SS
Salmacina dysteri	Tube forming worm	Towed video	10 frame grabs	Count	Individuals m^{-2}	-	SS
Scyliorhinus canicula	Lesser spotted catshark	Baited video	30 minute BRUV sample	Count	MaxN	-	M
Scyliorhinus stellaris	Nursehound	Baited video	30 minute BRUV sample	Count	MaxN	-	M
Sepia officinalis	Common cuttlefish	Baited video	30 minute BRUV sample	Count	MaxN	-	M
Serpula vermicularis	A tubeworm	Towed video	10 frame grabs	Count	Individuals m^{-2}	-	SS
'Shotgun' bivalve	'Shotgun' bivalve	Towed video	10 frame grabs	Count	Individuals m^{-2}	-	SS
Small white grouped sponges	Small white grouped sponges	Towed video	10 frame grabs	Count	Individuals m^{-2}	-	SS
Solea solea	Sole	Baited video	30 minute BRUV sample	Count	MaxN	-	M
Spondyliosoma cantharus	Black seabream	Baited video	30 minute BRUV sample	Count	MaxN	-	M
Styela clava	Stalked sea squirt	Towed video	10 frame grabs	Count	Individuals m^{-2}	-	RB
Sycon ciliatum	A solitary sponge	Towed video	10 frame grabs	Count	Individuals m^{-2}	-	RB
Symphodus bailloni	Baillon's Wrasse	Baited video	30 minute BRUV sample	Count	MaxN	-	M
Symphodus melops	Corkwing wrasse	Baited video	30 minute BRUV sample	Count	MaxN	-	M
Tethya aurantium	Golf ball sponge	Towed video	10 frame grabs	Count	Individuals m^{-2}	-	RB
Thyone fusus	A burrowing sea cucumber	Towed video	10 frame grabs	Count	Individuals m^{-2}	-	SS
Trachurus trachurus	Horse mackerel ('scad')	Baited video	30 minute BRUV sample	Count	MaxN	-	M
Trisopterus luscus	Pouting	Baited video	30 minute BRUV sample	Count	MaxN	-	M
Trisopterus minutus	Poor Cod	Baited video	30 minute BRUV sample	Count	MaxN	✓	M
Unidentified juvenile fish	Juvenile fish	Baited video	30 minute BRUV sample	Count	MaxN	-	M
Urticina felina	Dahlia anemone	Towed video	10 frame grabs	Count	Individuals m^{-2}	-	SS
Xantho hydrophilus	Montagu's crab	Baited video	30 minute BRUV sample	Count	MaxN	-	M
Zeugopteruspunctatus	Top knot	Baited video	30 minute BRUV sample	Count	MaxN	-	M
Zeus faber	John Dory	Baited video	30 minute BRUV sample	Count	MaxN	-	M

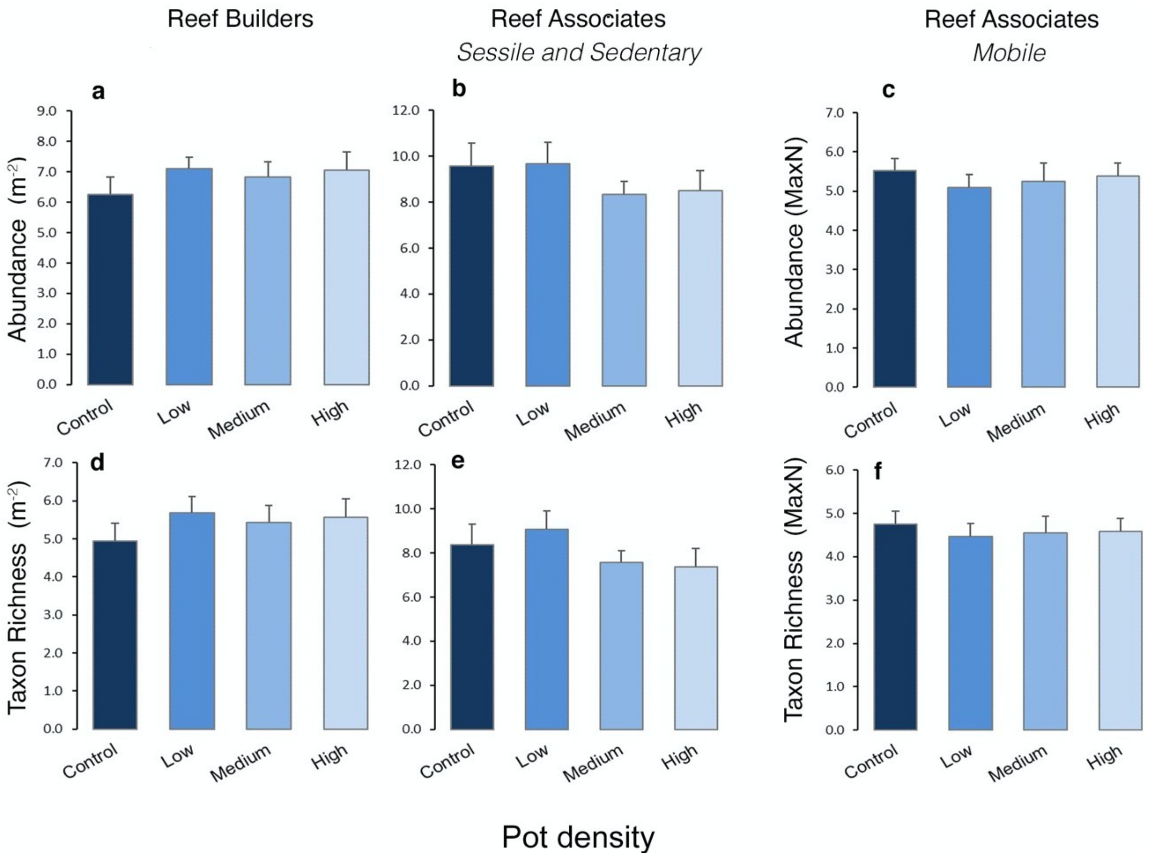

Supplementary Figure 1. Response variables for each functional Group by pot density Treatment; 2014 data. a, b, c Mean Abundance and **d, e, f** Taxon Richness (PERMANOVA main results from Supplementary Table 1), + standard error of the mean, for **a & d** grouped Reef Builders, **b & e** Sessile and Sedentary Reef Associates and **c & f** Mobile Reef Associates, for each pot density Treatment in 2014.

Supplementary Table 2. PERMANOVA main test results on fourth-root transformed data for differences between factors Year (fixed: 2014, 2017) Treatment (fixed: Control, Low Medium, High) and Area (random: Axmouth, Beer, Lyme Regis, West Bay) for Reef Builders; Taxon Richness, Reef Associates (Sessile and Sedentary, Mobile); Abundance and Taxon Richness. Degrees of freedom (df), Sum of Squares (SS), *F* and *P* are reported

**Group**	**Response variable**	**Model**				
Reef Builders	Taxon Richness	Source	df	SS	Pseudo-*F*	*P* (perm)
		Year (Ye)	1	1078.9	3.0305	0.1255
		Treatment (Tr)	3	1643.3	2.5276	0.1678
		Area (Ar)	3	1535.2	1.0434	0.4166
		Ye x Tr	3	1017.3	0.2918	0.889
		Ye x Ar	3	1068.1	1.1976	0.3044
		Tr x Ar	9	4413.9	1.6497	**0.0374**
		Residual	105	42285		
		Total	127	53042		
Reef Associates (Sessile and Sedentary)	Abundance	Source	df	SS	Pseudo-*F*	*P* (perm)
		Year (Ye)	1	3929.1	2.7982	0.2033
		Treatment (Tr)	3	3220.2	1.4023	0.2883
		Area (Ar)	3	3697.9	4.4988	0.2842
		Ye x Tr	3	2274.9	1.8706	0.1822
		Ye x Ar	3	4212.5	2.1035	0.3024
		Tr x Ar	9	2889.4	2.236	0.195
		Residual	105	44498		
		Total	127	64722		
Reef Associates (Sessile and Sedentary)	Taxon Richness	Source	df	SS	Pseudo-*F*	*P* (perm)
		Year (Ye)	1	1912.1	1.4414	0.3002
		Treatment (Tr)	3	2515.2	0.9427	0.4557
		Area (Ar)	3	3031.6	1.0184	0.4169
		Ye x Tr	3	1764.3	1.7459	0.1962
		Ye x Ar	3	2969.6	1.0107	0.2879
		Tr x Ar	9	4004.2	2.6889	0.362
		Residual	105	47148		
		Total	127	63345		
Reef Associates (Mobile)	Abundance	Source	df	SS	Pseudo-*F*	*P* (perm)
		Year (Ye)	1	3929.1	2.7982	1.945
		Treatment (Tr)	3	3220.2	1.4023	0.2895
		Area (Ar)	3	2879.2	1.5169	0.0782
		Ye x Tr	3	2274.9	1.8706	1.1805
		Ye x Ar	3	3648.4	1.1847	0.281
		Tr x Ar	9	4889.1	2.237	0.072
		Residual	105	41001		
		Total	127	64722		

Reef Associates (Mobile)	Taxon Richness	Source	df	SS	Pseudo-*F*	*P* (perm)
		Year (Ye)	1	1912.1	1.4414	0.3004
		Treatment				
		(Tr)	3	3515.2	0.1624	0.9149
		Area (Ar)	3	16624	1.525	0.1722
		Ye x Tr	3	1764.3	1.7459	0.2013
		Ye x Ar	3	3031.6	1.0184	0.4217
		Ye x Tr	9	2193.1	1.3267	0.2738
		Residual	105	49266		
		Total	127	63345		

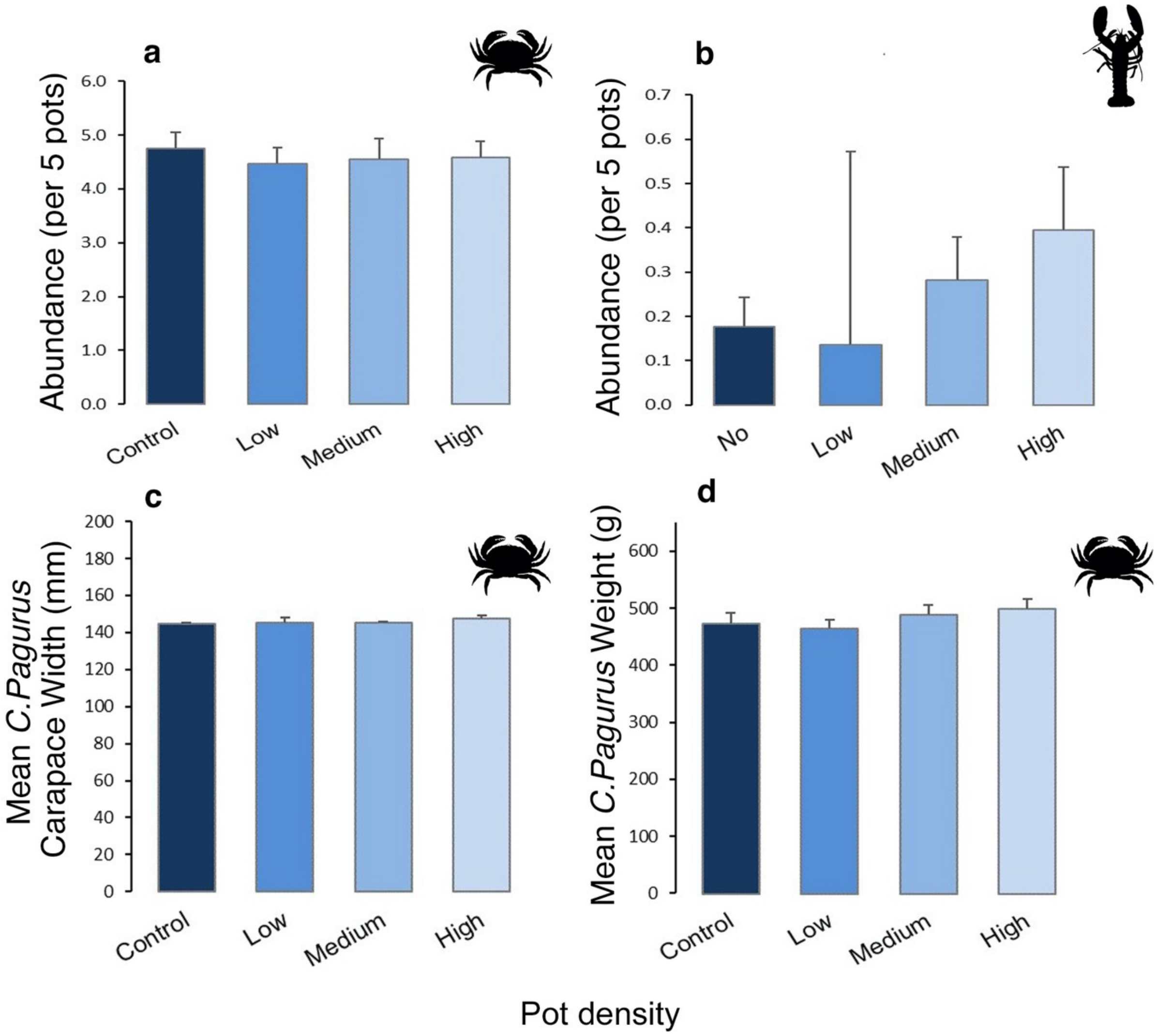

Supplementary Figure 2. Abundances of commercially targeted species, and brown crab (*Cancer pagurus)* biometric response variables, by Treatment (pot density); 2014 data. a & b Mean Abundance (PERMANOVA main results from Supplementary Table 2), + standard error of the mean, for **a** *Cancer pagurus* (brown crab) (Control (No) n = 494, Low n = 422, Medium n = 471, High n = 531) and **b** *Homarus gammarus* (European lobster) (No n = 38, Low n = 13, Medium n = 27, High n = 18) by pot density treatment. **c** Mean *C. pagurus* Carapace Width and **d** Mean *C. pagurus* Weight by pot density treatment (+ standard error of the mean)

Supplementary Table 3. PERMANOVA main test results on fourth-root transformed data for response variable Abundance of selected Indicator taxa between factors Year (fixed: 2014, 2017), Treatment (fixed: Control, Low Medium, High) and Area (random: Axmouth, Beer, Lyme Regis, West Bay). Degrees of freedom (df), Sum of Squares (SS), *F* and *P* are reported

**Indicator**	**Response variable**	**Model**				
Eunicella verrucosa	Abundance	Source	df	SS	Pseudo-F	P (perm)
		Year (Ye)	1	1.2891	1.387	0.827
		Treatment (Tr)	3	1.1591	1.2332	0.4309
		Area (Ar)	3	1.5059	0.8353	0.172
		Ye x Tr	3	1.2429	9.9796	0.0666
		Ye x Ar	3	0.7348	1.8955	0.2028
		Tr x Ar	9	1.8198	1.3321	0.232
		Residual	105	21.144		
		Total	127	28.896		
Asterias rubens	Abundance	Source	Df	SS	Pseudo-F	P (perm)
		Year (Ye)	1	1.9238	1.392	0.2378
		Treatment (Tr)	3	1.2938	0.357	0.823
		Area (Ar)	3	0.3478	1.129	0.192
		Ye x Tr	3	1.9283	1.2389	0.238
		Ye x Ar	3	0.347	1.239	0.687
		Tr x Ar	9	1.239	0.3289	0.123
		Residual	105	28.01		
		Total	127	35.09		
Grouped large anemones	Abundance	Source	df	SS	Pseudo-F	P (perm)
		Year (Ye)	1	0.5885	0.5375	0.5209
		Treatment (Tr)	3	1.3248	0.4837	0.7288
		Area (Ar)	3	1.4518	1.2288	0.2567
		Ye x Tr	3	1.8658	1.1927	0.3693
		Ye x Ar	3	0.3284	0.3858	0.7658
		Tr x Ar	9	1.584	1.289	0.293
		Residual	105	32.084		
		Total	127	39.227		
Chaetopterus variopedatus	Abundance	Source	df	SS	Pseudo-F	P (perm)
		Year (Ye)	1	2.665	1.6785	0.327
		Treatment (Tr)	3	1.3138	1.1368	0.0931
		Area (Ar)	3	1.184	0.382	0.832
		Ye x Tr	3	1.1828	1.0923	0.123
		Ye x Ar	3	0.8923	0.2893	0.2783
		Tr x Ar	9	0.237	0.29283	0.4785

		Residual	105	39.724		
		Total	127	47.199		
Trisopterus minutus	Abundance	Source	df	SS	Pseudo-F	P (perm)
		Year (Ye)	1	1.328	1,9283	0.7832
		Treatment (Tr)	3	0.2387	0.4289	0.982
		Area (Ar)	3	1.932	0.328	0.462
		Ye x Tr	3	1.4986	1.043	0.326
		Ye x Ar	3	1.0293	0.9835	0.4278
		Tr x Ar	9	1.8415	1.658	0.2378
		Residual	105	36.055		
		Total	127	43.923		
Labrus bergylta	Abundance	Source	df	SS	Pseudo-F	P (perm)
		Year (Ye)	1	1.0932	1.2438	0.6485
		Treatment (Tr)	3	0.9238	0.238	0.3598
		Area (Ar)	3	0.9123	0.7832	0.486
		Ye x Tr	3	0.2384	0.875	0.2148
		Ye x Ar	3	0.128	0.4986	0.287
		Tr x Ar	9	4.923	0.4786	0.832
		Residual	105	8.219		
		Total	127	14.343		
Necora puber	Abundance	Source	df	SS	Pseudo-F	P (perm)
		Year (Ye)	1	0.578	0.586	0.3489
		Treatment (Tr)	3	0.5687	0.6587	0.3489
		Area (Ar)	3	1.9324	1.7845	0.173
		Ye x Tr	3	0.238	0.3875	0.457
		Ye x Ar	3	0.4785	0.547	0.468
		Tr x Ar	1	1.847	1.0945	0.359
		Residual	105	17.246		
		Total	127	22.889		
